# Meta-analysis comparing chewing gum versus standard postoperative care after colorectal resection

**DOI:** 10.18632/oncotarget.11735

**Published:** 2016-08-31

**Authors:** Guo-Min Song, Yong-Hong Deng, Ying-Hui Jin, Jian-Guo Zhou, Xu Tian

**Affiliations:** ^1^ Department of Nursing, Tianjin Hospital, Tianjin 300211, China; ^2^ School of Nursing, Tianjin University of Traditional Chinese Medicine, Tianjin 300193, China; ^3^ Evidence-Based Nursing Center, Tianjin University of Traditional Chinese Medicine, Tianjin 300193, China; ^4^ Department of Oncology, Affiliated Hospital of Zunyi Medical University, Zunyi 563000, China; ^5^ Department of Nursing, Chongqing Cancer Institute, Chongqing 400020, China

**Keywords:** chewing gum, postoperative ileus, colorectal surgery, meta-analysis, trial sequential analysis

## Abstract

**Background:**

Previous incomplete studies investigating the potential of chewing gum (CG) in patients undergoing colorectal resection did not obtain definitive conclusions. This updated meta-analysis was therefore conducted to evaluate the effect and safety of CG versus standard postoperative care protocols (SPCPs) after colorectal surgery.

**Results:**

Total 26 RCTs enrolling 2214 patients were included in this study. The CG can be well-tolerated by all patients. Compared with SPCPs, CG was associated with shorter time to first flatus (weighted mean difference (WMD) −12.14 (95 per cent c.i. −15.71 to −8.56) hours; P < 0.001), bowl movement (WMD −17.32 (−23.41 to −11.22) hours; P < 0.001), bowel sounds (WMD −6.02 (−7.42 to −4.63) hours; P < 0.001), and length of hospital stay (WMD −0.95 (−1.55 to −0.35) days; P < 0.001), a lower risk of postoperative ileus (risk ratio (RR) 0.61 (0.44 to 0.83); P = 0.002), net beneficial and quality of life. There were no significant differences between the two groups in overall complications, nausea, vomiting, bloating, wound infection, bleeding, dehiscence, readmission, reoperation, mortality.

**Materials and Methods:**

The potentially eligible randomized controlled trials (RCTs) that compared CG with SPCPs for colorectal resection were searched in PubMed, Embase, Cochrane library, China National Knowledge Infrastructure (CNKI), and Chinese Wanfang databases through May 2016. The trial sequential analysis was adopted to examine whether a firm conclusion for specific outcome can be drawn.

**Conclusions:**

CG is benefit for enhancing return of gastrointestinal function after colorectal resection, and may be associated with lower risk of postoperative ileus.

## INTRODUCTION

Postoperative ileus is an important complication after colorectal surgery, which is characterized mainly by nausea, vomiting and abdominal distension [1]. It is associated with delayed postoperative recovery, prolonged length of hospital stay (LOS) and increased healthcare costs [2]. In the United States, the annual medical expenditures of managing postoperative ileus have been estimated to be 1 billion dollars [3]. Accordingly, it is quite important to prevent and reduce this given condition. To date, many methods (e.g. fluid restriction, early mobilization and nutrition) have been increasingly investigated in order to alleviate postoperative ileus [4–6], of which chewing gum (CG) has become a promising option. However, the efficacy of CG for patients after colorectal surgery is still debatable [1, 7, 8].

Previous systematic reviews [8–18] have been performed to address this issue, and shown that CG may be a safe and inexpensive approach to enhance return of intestinal function following colorectal resection and shorten the LOS. However, little attention had been paid on several important outcomes including time to first bowel sounds, time to first feeding, mortality, economic effect and quality of life (QoL) in these incomplete analyses [8–18]. Furthermore, several randomized controlled trials (RCTs) with moderate or large sample size, which investigated the comparative efficacy of CG versus standard postoperative care protocols (SPCPs) in patients undergoing colorectal resection, have been published recently.

Considering these aspects, we therefore undertaken this updated meta-analysis to comprehensively evaluate the effect and safety of CG versus SPCPs for patients undergoing colorectal resection.

## RESULTS

### Literature search

**Figure 1:**
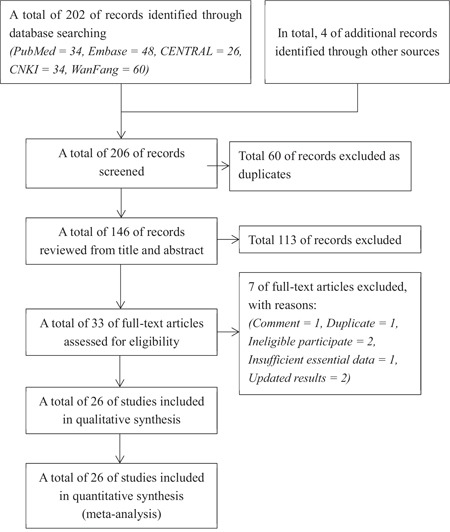
The flow diagram of identification and selection of studies for the present meta-analysis CENTRAL = Cochrane Central Registry for Controlled trials, CNKI = China National Knowledge Infrastructure.

The identification and selection of studies was graphically depicted in Figure 1. Electronic database searches captured 202 records, and 4 records were obtained from reference lists of relevant reviews. Sixty duplicate records were removed, and 113 records were eliminated by checking the titles and abstracts. The remaining 33 full-text articles were assessed for eligibility. After application of the inclusion criteria, a total of 26 studies [7, 19–43] comprising 2214 participants were eligible for our inclusion criteria and included to perform meta-analysis.

### Study characteristics

Study characteristics of included studies were documented in Table 1. Of which 17 [21, 22, 24–26, 28–39] recruited the patients undergoing open colorectal surgery, 7 [7, 20, 23, 27, 41–43] performed hybrid surgery approaches (open and laparoscopic), and 2 [19, 40] conducted laparoscopic surgery. Of these 26 studies, it is noted that, 3 [41–43] were conference abstract, and the corresponding information were extracted from the Cochrane systematic review published by Short et al. [14]. All studies were published between 2002 and 2016, and the sample size in individual trials ranged from 19 to 402.

**Table 1:** Basic characteristics of all eligible included in the present meta-analysis

References	Country	NO. of patients (GCG/CG)	Men (%)	Age (years) (GCG/CG)	Interventions		Article type	Outcomes
					GCG	CG		
Asao *et al*. (2002)^31^	Japan	19 (10/9)	68.4	58.6/60.6	Sugarless chewing gum, 3 times a day, from the first postoperative morning until the day patients began oral intake.	The same route postoperative care as the intervention group, excluding gum chewing.	Full text	TFF, TFBM, LOS, complications, TCG.
Atkinson *et al*. (2016)^32^	UK	402(199/203)	57.1	65.5/66.9	Sugarless chewing gum, at least 10 min every time, 4 times a day, from the first postoperative morning for 5 days, plus usual care (ERAS).	The same usual care (ERAS) as the intervention group, excluding gum chewing.	Full text	TFF, TFBM, LOS, TFBS, complications, economic effect, QoL.
Bahena-Aponte *et al*. (2010)^33^	Mexico	32(16/16)	62.5	55.6/56.6	Sugarless chewing gum, 30 min every time, 3 times a day, from immediately postoperatively (within 24 h) until patients tolerated oral intake.	The same standard postoperative care as the intervention group, except the chewing gum.	Full text	TFF, TFBM, LOS, complications.
Bonventre *et al*. (2014)^34^	Italy	50(25/25)	UC	UC	Sugarless peppermint flavoured gum, 30 min every time, 3 times a day, starting from 6 h postoperatively.	The same route postoperative care as the intervention group, excluding gum chewing.	Full text	TFF, TFBM, LOS, complications, TCG.
Forrester *et al*. (2014)^35^	America	30(13/17)	33.3	55.8/63.3	Sugarless chewing gum, at least 60 min every time, at least 3 times a day, from the first postoperative morning or after removal of the nasogastric tube.	The same standard postoperative care as the intervention group, excluding gum chewing.	Full text	TFF, TFBM, LOS, complications.
Heijkant *et al*. (2015)^36^	Netherlands	112(52/60)	75.0	66.0/67.0	Sugarless gum, average 24 pieces, starting from 3 h before the start of the operation and 3 h after surgery respectively.	Patients received a dermal patch 3 h before surgery but they were instructed not to chew gum.	Full text	LOS, complications.
Hirayama *et al*. (2006)^37^	Japan	24(10/14)	41.7	55.6/60.6	Commercial sugarless gum, about 30 min every time, 3 times a day, from the first postoperative morning.	The same medical care as the intervention group, excluding gum chewing.	Full text	TFF, TFBM, complications.
Kobayashi *et al*. (2015)^38^	Japan	43(21/22)	60.5	66.4/68.0	Commercial blueberry-flavored nonxylitol gum, at least 5 minutes every time, 3 times a day, from postoperative day 1 to the first day of food intake.	The same medical care as the intervention group, excluding gum chewing.	Full text	TFF, TFBM, LOS, complications.
Lim *et al*. (2013)^39^	Australia	157(77/80)	60.5	63.0/62.0	Sorbitol-free gum, 15 min every time, 4 times a day, plus established ERAS program.	The same medical care as the intervention group, excluding gum chewing.	Full text	TFF, TFBM, LOS, complications, TCG.
Matros *et al*. (2006)^40^	America	43(22/21)	46.5	62.0/58.0	Sugarless peppermint-flavoured gum, 45 min every time, 3 times daily until passage of flatus.	The same medical care as the intervention group, excluding gum chewing.	Full text	TFF, TFBM, LOS, complications.
Quah *et al*. (2006)^41^	UK	38(19/19)	65.8	67.0/68.0	Sugarless chewing gum, least 5 min every time, 3 times daily, from the first postoperative morning until the oral intake of a solid diet.	The same medical care as the intervention group, excluding gum chewing.	Full text	TFF, TFBM, LOS, complications, TCG.
Schuster *et al*. (2006)^42^	America	34(17/17)	67.6	60.0/63.0	Sugarless chewing gum, 60 min every time, 3 times daily, until discharge.	The same medical care as the intervention group, excluding gum chewing.	Full text	TFF, TFBM, LOS, complications, TCG.
Topcu *et al*. (2016)^43^	Turkey	60(30/30)	50.0	63.9 (overall)	Chewing gum, 15 min every time, 3 times a day, from the first morning after the operation until the time of their discharge.	The same medical care as the intervention group, excluding gum chewing.	Full text	TFF, TFBM, LOS.
Zaghiyan *et al*. (2013)^7^	America	114(54/60)	58.8	42.1/48.8	Sugared chewing gum, 45 min every time, 3 times a day, plus ERAS program.	The same medical care as the intervention group, excluding gum chewing.	Full text	TFF, TFBM, LOS, complications.
Cao *et al*. (2008)^44^	China	115(58/57)	53.9	53.0 (0verall)	Sugarless chewing gum, 15min every time, 3 times a day, from 12 to 24 h postoperatively until first flatus.	The same medical care as the intervention group, excluding gum chewing.	Full text	TFF, complications.
Fan *et al*. (2009)^45^	China	42(21/21)	61,9	47.6/49.7	Xylitol sugarless gum, 30 min every time, 3 times a day, from the first postoperative day until they were asked to stop fasting.	The same medical care as the intervention group, excluding gum chewing.	Full text	TFF, TFBM, LOS.
Li *et al*. (2012)^46^	China	73(38/35)	58.9	54.3/56.2	Xylitol sugarless chewing gum, 15 to 20 min every time, from 8 h after surgery until bowel exhaustion.	The same medical care as the intervention group, excluding gum chewing.	Full text	TFF, TFBM, LOS.
Tian *et al*. (2013)^47^	China	100(50/50)	56.0	52.1/53.9	Extra sugarless chewing gum, 15 to 20 min every time, 4 to 5 times a day, from 2 to 4 h after surgery until first flatus or first bowel movement.	The same medical care as the intervention group, excluding gum chewing.	Full text	TFF, TFBM, complications.
Wang *et al*. (2011)^48^	China	155(78/77)	65.2	55.6/52.6	Chewing gum, 15 min every time, 1 time every 4 h in day time, from 6 h postoperatively until the first postoperative exhaustion.	The same medical care as the intervention group, excluding gum chewing.	Full text	TFF, TFBM, LOS, TFBS, complications, TCG.
Wang *et al*. (2016)^49^	China	110(76/34)	u.c.	u.c.	Commercially available chewing gum, 15 to 20 min every time, 2 times a day, from the 8h after surgery until the day they return to first flatus.	The same medical care as the intervention group, excluding gum chewing.	Full text	TFF, TFBS, complications.
Zhang *et al*. (2015)^50^	China	104(52/52)	57.7	48.5/48.3	Chewing gum, 5 to 10 min every time, 6 times a day, from 1h after wakefulness from anesthesia to the time of first flatus.	The same medical care as the intervention group, excluding gum chewing.	Full text	TFF, TFBM, TFBS, complications.
Zhong *et al*. (2009)^51^	China	120(60/60)	u.c.	u.c.	Sugarless chewing gum, 5 to 25 min every time, 3 times a day, from 12 h after surgery.	The same medical care as the intervention group, excluding gum chewing.	Full text	TFF, TFBM, LOS, complications.
McCormick *et al*. (2005)^53^	America	102(62/40)	u.c.	60.0 (overall)	Chewing gum, 15 min every time, 4 times a day.	The same medical care as the intervention group, excluding gum chewing.	Abstract^§^	TFF, TFBM, LOS, TCG.
Crainic *et al*. (2009)^52^	Portland	44(20/24)	27.8	u.c.	Extra sugarless chewing gum, 30 min every time, 3 times a day, from within 24 h until first bowel movement.	The same medical care as the intervention group, excluding gum chewing.	Full text	TFF, TFBM.
Schluender *et al*. (2006)^54^	America	38(17/19)	42.9	u.c.	Sugarless chewing gum, at least 30 min every time, 3 times a day, from postoperative day 1 throughout their hospital stay.	The same medical care as the intervention group, excluding gum chewing.	Abstract^§^	TFF, TFBM, LOS, complications.
Watson *et al*. (2008)^55^	UK	53(26/27)	47.4	70.6/69.2	Sugarless chewing gum, 30 min every time, 3 times a day, from the first postoperative morning until day of discharge.	The same medical care as the intervention group, excluding gum chewing.	Abstract^§^	TFF, TFBM, LOS, complications, TCG.

GCG, gum-chewing group, CG, control group;

§ Conference abstract (sufficient data obtained from the Cochrane systematic review by Short and colleagues); u.c., unclear; TFF, time to first flatus; TFMB, time to first bowel movement; LOS, length of hospital stay; TFBS, time to first sounds; TCG, tolerability of chewing gum, QoL, quality of life.

### Risk of bias

**Figure 2:**
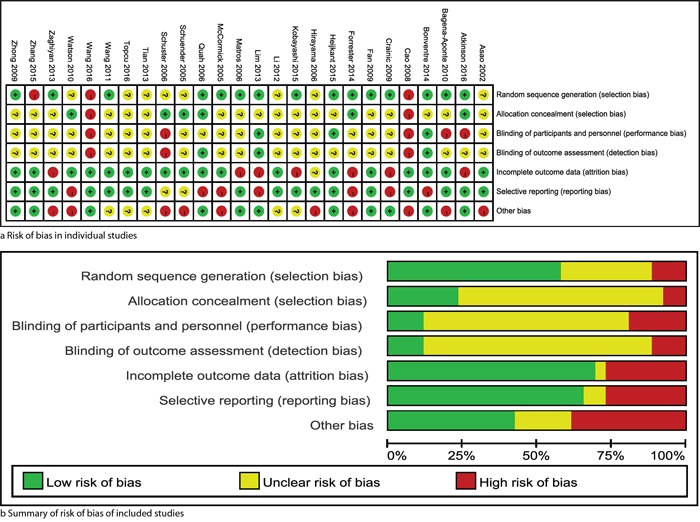
Risk of bias: risk of bias in individual trials **a.** and summary of brisk of bias of included studies **b.** The yellow, green and red represent “unclear risk of bias”, “low risk of bias” and “high risk of bias” respectively.

Details of risk of bias for individual trials were exhibited in Figure 2a, and the summary of risk of bias of included studies in Figure 2b. Most studies [7, 20–24, 27–29, 33, 36, 39, 40] generated appropriately the random sequence, and 6 studies [20, 23, 29, 30, 42, 43] conducted adequately allocation concealment. Because it is extremely difficult to blind the participants and surgeons, almost all of these studies were valued as unclear risk of bias for this domain.

### Level of evidence

We documented the GRADE evidence profile for all outcomes in Supplementary Table S1. In this systematic review, we assessed 20 outcomes, of which time to first flatus, bowel movement, bowel sounds, feeding, and postoperative ileus were listed as critical outcomes and remaining 15 outcomes such as LOS and overall complications were viewed as important outcomes. The level of evidence was moderate for time to first bowel sounds and complications related to CG, while the level of evidence for remaining outcomes was low or very low.

### Outcome

#### Primary outcome

**Figure 3:**
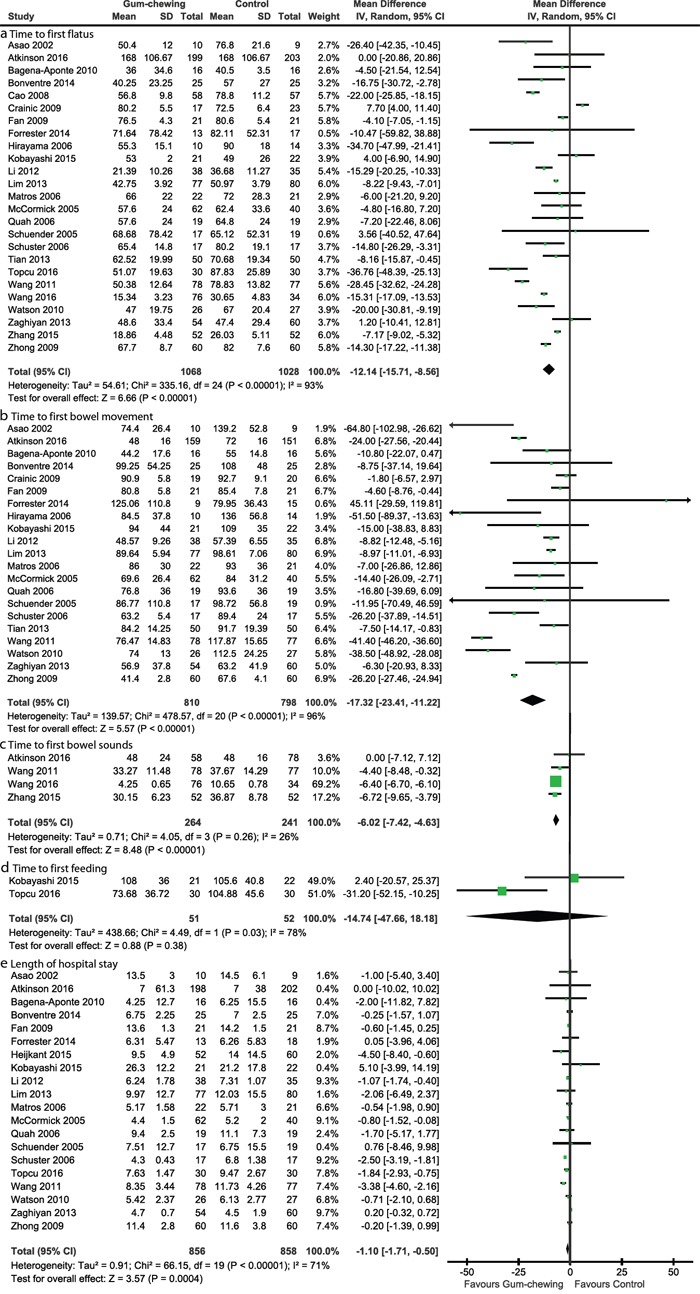
Forest plots of primary outcomes: time to first flatus **a.**, time to first bowel movement **b.**, time to first bowel sounds **c.**, time to first feeding **d.**, and length of hospital stay **e.** The summary effect estimate (weighted mean difference, WMD) for individual randomized controlled trials (RCTs) are indicated by green rectangles (the size of the rectangle is proportional to the study weight), with the black horizontal lines representing 95 per cent c.i. The overall summary effect estimate and 95 per cent c.i. are indicated by the black diamond below. SD = standard deviation, IV = inverse variance.

Pooled results indicated that CG significantly reduced the time to first flatus (WMD −12.14, (95 per cent c.i. −15.71 to −8.56) hours; P < 0.001) (I2 = 93 per cent), time to first bowel movement (WMD −17.32, (95 per cent c.i. −23.41 to −11.22) hours; P < 0.001) (I2 = 96 per cent), time to first bowel sounds (WMD −6.02, (95 per cent c.i. −7.42 to −4.63) hours; P < 0.001) (I2 = 26 per cent), and LOS (WMD −0.95, (95 per cent c.i. −1.55 to −0.35) days, P < 0.001) (I2 = 71 per cent), but no significant difference in time to first feeding between CG and SPCPs groups (WMD −14.74, (95 per cent c.i. −47.66 to 18.18) hours; P = 0.38) (I2 = 78 per cent) (Figure 3). We also examined the robust of pooled results through excluding conference abstract and studies with less than 20 patients per arm respectively, and shown that all results did not changed significantly (Supplementary Figure S1, Supporting Information).

For time to first flatus, time to first bowel movement, time to first bowel sounds, and LOS, the accumulative Z-curve crossed the sequential monitory boundary, which suggested that the firm conclusions can be drawn based on the present accumulated information size and additional resources should not be wasted to plan further studies (Supplementary Figure S2, Supporting Information). For time to first feeding, trial sequential analysis was not performed due to the fact that finite studies (only 2 trials) were accrued, and thus further studies may be warranted to detect the difference between CG and SPCPs.

#### Secondary outcomes

CG significantly reduced the risk of postoperative ileus (RR 0.61, 95 per cent c.i. 0.44 to 0.83; P = 0.002) (I2 = 0 per cent), but did not decrease the risk of remaining secondary outcomes compared to SPCPs (Supplementary Figure S3, Supporting Information). The tolerability of CG was reported in 8 studies [19, 22, 27, 29, 30, 36, 41, 43], and the qualitative findings suggested that CG was well-tolerated by all patients in active group. Atkinson et al.' study [20] reported economic effect and QoL, and the results suggested that patients who were informed to consume CG had a lower net benefit (MD – €173, 95 per cent c.i. – €1103 to 757) and worse QoL (no difference on day after operation 4 days, but worse at 6 and 12 weeks).

For postoperative ileus, the accumulative Z-curve crossed the conventional monitory boundary, but did not surpass the sequential monitory boundary, which suggested that a false positive conclusion was generated result from chance (Supplementary Figure S4, Supporting Information). For remaining secondary outcomes, the accumulative Z-curve did not cross the conventional or sequential monitory boundaries (Supplementary Figure S4), which suggested that the accumulative information size too insufficient power to draw firm conclusions.

### Subgroup analysis

We conducted subgroup analyses for outcomes with extremely heterogeneity according to the type of surgical approaches, and shown that CG decreased significantly time to first flatus in patients underwent laparoscopic and open colectomy, time to first bowel movement in all patients received various surgical approaches, and time to first bowel sounds, LOS and postoperative ileus in patients received open colectomy. The details of subgroup analyses were documented in Supplementary Table S2.

It is noted that, however, all significant heterogeneities were not decreased or omitted due to implementation of subgroup analyses. After carefully reviewed included trials, we detected a fact that all included trial generated different coverages due to incorporation of trials with large and small sample size in the present systematic review. And because of this, we confound these extremely heterogeneities. However, we can easily found that the effect estimates with consistent direction were generated by most included trials in each meta-analysis on outcome of interest, and thus we can reasonably establish that these extremely heterogeneities cannot impair the robust and validity of corresponding summary effect size.

### Publication bias

The funnel plots, which often be drawn to inspect the existence of publication bias, were depicted in Supplementary Figure S5 (Supporting Information). For most primary (time to first flatus, time to first bowel movement and length of hospital stay) and secondary outcomes, the asymmetric funnel plots were constructed, which indicated the existence of publication bias.

## DISCUSSION

### Main findings

Our meta-analysis of 26 RCTs involving 2214 patients suggested that CG was well-tolerated by all patients in active group, and significantly decreased the time to first flatus, time to first bowel movement, time to first bowel sounds, and shortened LOS compared with SPCPs. Furthermore, the patients were informed to consume CG may experience the lower risk of postoperative ileus.

### Comparison with other studies

In order to determine the effect and safety of CG in patients undergoing colorectal surgery, 11 meta-analyses [8–18] have been published previously. The characteristics and outcomes of these all meta-analyses have been summarized in Table 2.

**Table 2:** Meta-analyses of gum-chewing versus conventional postoperative care for patients underwent colorectal resection

Items	Chan *et al*. (2007)^9^	de Castro *et al*. (2008)^10^	Purkayastha *et al*. (2008)^8^	Parnaby *et al*. (2009)^13^	Vásquez *et al*. (2009)^15^	Li *et al*. (2013)^12^	Wang *et al*. (2013)^16^	Yin *et al*. (2013)^18^	Ho *et al*. (2014)^11^	Short *et al*. (2015)^14^	Yang *et al*. (2015)^17^	Present study
NO. of RCTs pooled	5	5	5	6	6	8	13	7	10	22	9	26
NO. of patients	158	158	158	256	244	322	993	278	271	1668	686	2214
Search strategy until (year)	January, 2007	June, 2007	July, 2006	July, 2008	August, 2008	December, 2012	April, 2013	February, 2012	April, 2013	June, 2014	December, 2014	May, 2016
Restriction imposed												
Publication language	Yes	Yes	No	No	n.r.	No	n.r.	n.r.	No	No	Yes	No
Publication status	n.r.	Yes	n.r.	n.r.	Yes	No	n.r.	Yes	No	No	n.r.	No
Outcomes												
Time to first flatus (h)	WMD −20.78 (−32.64, −8.93)	WMD −19.30 (−30.19, −8.42)	WMD −15.84 (−26.64, −4.8)	WMD −0.54 (−18.96, −6.96)	WMD −14.00 (−23.45, −4.55)	WMD −7.20 (−15.64, 1.92)	WMD −11.66 (−17.26, −6.07)	WMD −14.69 (−24.67, −4.70)	SMD −0.52 (−0.86, −0.18)	WMD −12.46 (−17.17, −7.76)	WMD −17.33 (−23.96, −10.70)	WMD −12.14 (−15.71, −8.56)
Time to first bowel movement (h)	WMD −33.25 (−50.80, −15.70)	WMD −29.67 (−46.03, −13.32)	WMD −26.64 (−42.96, −10.08)	WMD −0.67 (−30.24, −2.64)	WMD −24.99 (−42.31, −7.66)	WMD −17.76 (−32.88, −2.64)	WMD −32.31 (−56.89, −7.73)	WMD −18.76 (−25.07, −12.44)	SMD −0.50 (−0.99, −0.01)	WMD −18.09 (−25.32, −10.85)	WMD −22.25 (−36.45, −8.05)	WMD −17.32 (−23.41, −11.22)
Time to first bowel sounds (h)	n.r.	n.r.	n.r.	n.r.	n.r.	n.r.	n.r.	n.r.	n.r.	WMD −3.21 (−7.04, 0.62)	n.r.	WMD −6.02 (−7.42, −4.63)
Time to first feeding (h)	n.r.	n.r.	n.r.	n.r.	n.r.	n.r.	n.r.	n.r.	n.r.	n.r.	n.r.	WMD −14.74 (−47.66, 18.18)
Length of hospital stay (days)	WMD −2.44 (−3.10, −1.77)	WMD −1.26 (−3.16, 0.64)	WMD −1.25 (−3.27, 0.77)	No difference (*P* = 0.33)	WMD −26.17 (−57.51, 5.18)	WMD −1.10 (−2.37, 0.17)	WMD −1.10 (−1.93, −0.27)	WMD −0.92 (−2.32, 0.47)	SMD −0.50 (−0.86, −0.14)	WMD −1.01 (−1.61, −0.41)	WMD −1.37 (−2.25, −0.49)	WMD −0.95 (−1.55, −0.35)
Overall complication rate	OR 0.45 (0.20, 1.00)	n.r.	n.r.	No difference (*P* = 0.324)	n.r.	n.r.	n.r.	n.r.	RR 0.69 (0.51, 0.93)	Not estimable	n.r.	RR 0.90 (0.69, 1.16)
Other complications	n.r.	n.r.	n.r.	n.r.	n.r.	n.r.	n.r.	n.r.	n.r.	GC lower than CC	n.r.	RR 1.05 (0.67, 1.63)
Postoperative ileus	OR 0.36 (0.07, 1.96)	n.r.	n.r.	n.r.	n.r.	n.r.	n.r.	n.r.	n.r.	Not estimable	OR 0.33 (0.14, 0.77)	RR 0.61 (0.44, 0.83)
Nausea	n.r.	n.r.	n.r.	n.r.	n.r.	n.r.	OR 0.89 (0.56, 1.40)	n.r.	n.r.	GC lower than CC	OR 0.73 (0.41, 1.07)	RR 1.01 (0.82, 1.23)
Vomiting	n.r.	n.r.	n.r.	n.r.	n.r.	n.r.	OR 0.90 (0.59, 1.38)	n.r.	n.r.	GC lower than CC	OR 0.80 (0.42, 1.52)	RR 1.01 (0.79, 1.28)
Bloating	n.r.	n.r.	n.r.	n.r.	n.r.	n.r.	OR 0.52 (0.35, 0.80)	n.r.	n.r.	Not estimable	OR 0.83 (0.24, 2.94)	RR 0.73 (0.49, 1.10)
Overall infections rate	n.r.	n.r.	n.r.	n.r.	n.r.	n.r.	n.r.	n.r.	n.r.	No difference	n.r.	RR 0.87 (0.50, 1.51)
Wound infection	OR 0.75 (0.17, 3.25)	n.r.	n.r.	n.r.	n.r.	n.r.	n.r.	n.r.	n.r.	Not estimable	n.r.	RR 0.80 (0.29, 2.20)
Other infections	n.r.	n.r.	n.r.	n.r.	n.r.	n.r.	n.r.	n.r.	n.r.	No difference	n.r.	RR 0.71 (0.35, 1.44)
Bleeding	OR 1.00 (0.13, 7.94)	n.r.	n.r.	n.r.	n.r.	n.r.	n.r.	n.r.	n.r.	Not estimable	n.r.	RR 0.95 (0.42, 2.16)
Wound dehiscence	OR 0.30 (0.01, 7.88)	n.r.	n.r.	n.r.	n.r.	n.r.	n.r.	n.r.	n.r.	Not estimable	n.r.	RR 1.12 (0.05, 23.25)
Anastomotic leak	Not estimable	n.r.	n.r.	n.r.	n.r.	n.r.	n.r.	n.r.	n.r.	Not estimable	n.r.	RR 0.72 (0.34, 1.57)
Complications related to GC	OR 0.53 (0.13, 2.19)	n.r.	n.r.	No occurrence	n.r.	n.r.	n.r.	n.r.	n.r.	No occurrence	n.r.	RR 3.06 (0.13, 74.67)
Readmission rate	OR 0.36 (0.07, 1.96)	n.r.	n.r.	n.r.	n.r.	n.r.	n.r.	n.r.	RR 0.81 (0.32, 2.02)	No difference	n.r.	RR 0.89 (0.35, 2.26)
Reoperation rate	OR 1.36 (0.08, 23.55)	n.r.	n.r.	n.r.	n.r.	n.r.	n.r.	n.r.	RR 0.85 (0.28, 2.59)	Not estimable	n.r.	RR 1.33 (0.09, 19.75)
Mortality	n.r.	n.r.	n.r.	n.r.	n.r.	n.r.	n.r.	n.r.	n.r.	No difference	n.r.	RR 1.57 (0.42, 5.92)
Tolerability of gum	n.r.	n.r.	n.r.	n.r.	n.r.	n.r.	n.r.	n.r.	n.r.	Well−tolerated	n.r.	Well−tolerated
Economic effect	n.r.	n.r.	n.r.	n.r.	n.r.	n.r.	n.r.	n.r.	n.r.	n.r.	n.r.	CC less than GC
Quality of life	n.r.	n.r.	n.r.	n.r.	n.r.	n.r.	n.r.	n.r.	n.r.	n.r.	n.r.	No difference on POD 4, CC better than GC at 6 and 12 weeks.
Trial sequential analysis	n.r.	n.r.	n.r.	n.r.	n.r.	n.r.	n.r.	n.r.	n.r.	n.r.	n.r.	Confirmed TFF, TFBM, TFBS, LOS.

Values in parentheses are 95 percent c.i.; RCT, randomized clinical trial; CENTRAL, Cochrane Central Register of Controlled Trials; CDSR, Cochrane Database of Systematic Review; DARE, Database of Abstracts of Reviews of Effects; CINAHL, cumulative index to nursing and allied health literature; WOS, Web of Science; CBM, China Biomedical Literature database; CNKI, China National Knowledge Infrastructure; WHO, world health organization; ICTRP, International Clinical Trials Registry Platform; n.r., not reported; GC, gum-chewing; CC, control group; TFF, time to first flatus; TFBM, time to first bowel movement; TFBS, time to first bowel sounds; LOS, length of hospital; POD, post-operation day; WMD, weighted mean difference; SMD, standard mean difference; OR, odds ratio; RR, risk ratio.

Of 11 previous meta-analyses, Chan et al. (2007) [9], de Castro et al. (2008) [10], and Purkayastha et al. (2008) [8] included 5 eligible RCTs comprising 158 patients to evaluate the potential of CG on the gastrointestinal function after colorectal resection, and found that CG significantly shortened the time to first flatus and time to first bowel movement, but the conclusions on LOS were controversial. Parnaby et al. (2009) [13], Vásquez et al. (2009) [15], Yin et al. (2013) [18], Li et al. (2013) [12], and Ho et al. (2014) [11] included 6 RCTs involving 256 patients, 6 RCTs involving 244 patients, 7 RCTs involving 278 patients, 8 RCTs involving 322 patients, and 10 RCTs involving 271 patients to conduct meta-analysis that compared the CG with SPCPs in colorectal surgery to carried out meta-analyses respectively. Of 5 meta-analyses, all [11–13, 15, 18] found that CG was associated with shorter time to first bowel movement, and 4 [11, 13, 15, 18] (excluding Li et al.'s study) suggested that CG significantly reduced time to first bowel movement, and 4 [12, 13, 15, 18] (excluding Ho et al.'s study) shown that patients who were informed to consume CG did not experience shorter LOS. It is noted that, however, a fatal limitation in these meta-analyses is that small sample size were accumulated. Small sample size is not enough power to draw a true inference due to chance (also termed as random error). Therefore, conclusions drawn from these meta-analyses may not be considered as definitive.

Three meta-analyses [14, 16, 17] with relatively large accumulated sample size (686, 993, and 1668 patients respectively) also evaluated the potential of CG on resumption of gastrointestinal function after colorectal surgery, and found that CG significantly decreased time to first flatus, time to first bowel movement, and LOS. However, these three meta-analyses [14, 16, 17] did not consider time to first feeding, economic effect and QoL as outcomes, which were useful for clinicians and policy makers. Furthermore, meta-analysis is an important technique to determine the magnitude and significance of an intervention, but these authors did not perform pooled quantitative analyses of most adverse outcomes (e.g. postoperative ileus and mortality). As a result, the use of CG in patients undergoing colorectal resection still remains controversial.

Compared with 11 previous meta-analyses [8–18], the present meta-analysis has several strengths. The period of our meta-analysis was until May 2016, which was longer than the other periods (the lasted study was until December 2014), and thus more studies and sample were accrued in our meta-analysis (26 RCTs involving 2214 patients). Language and publication status restrictions of the studies were not imposed in our study, and thus additional 2 Chinese studies (with 110 and 104 patients respectively) were captured. We quantitatively pooled data of those complications which sufficient information can be extracted, which may help clinicians to objectively assess the safety of CG. More outcomes were evaluated in our meta-analyses, and the effect and safety of CG can be more comprehensively assessed based on our pooled results. Furthermore, we adopted the trial sequential analysis method to determine whether further studies are warranted to detect differences between CG and SPCPs, and confirmed the evidence which CG significantly reduced the time to first flatus, time to first bowel movement, time to first bowel sounds, and LOS.

### Limitations

We must not fail to acknowledge the several limitations in our meta-analysis. First, substantial heterogeneity across studies was detected, which can perhaps be explained by a variety of colorectal pathologies and SPCPs in primary studies. Second, most trials in our study were rated as to be high risks of bias, which may overestimated the benefits and harms of CG. Third, we did not excluded the Zaghiyan et al.'s trial, in which patients in active group were informed to chewed sugared gum, because this two types of gum can produce similar effect [3]. Forth, limited data were available on economic effect of CG and QoL; the conclusions may be changed if further studies were added. Fifth, although we performed subgroup analyses according to the different surgery approaches, the significant heterogeneity in each outcome which has been explained to have no impact on robust and validity of corresponding pooled result was not significantly decreased or omitted, and thus further investigation on this issue are warranted. Sixth, we did not carry out additional analyses according to different quantities, frequencies, and durations of consuming CG, which may contribute to substantial heterogeneity.

## MATERIALS AND METHODS

We designed this meta-analysis according to the recommendations of Cochrane handbook for systematic reviews of interventions [44] and reported the pooled results in accordance with preferred reporting item for systematic review and meta-analysis statement (PRISMA) [45]. There was no formal protocol for this meta-analysis.

### Search strategy

We assigned two investigators (Y.-H.D. and X.T.) searched independently PubMed, Cochrane Central Register of Controlled Trials (CENTRAL), Embase, China National Knowledge Infrastructure (CNKI) and WanFang databases from inception to January 31, 2016, and the last search was updated on May 31, 2016. All search algorithms were structured using Exploded Medial Subject Heading and appropriate keywords, including “chew*”, “gum”, “colorectal”, “resection” and “random*”. No language and publication status were imposed. We also checked manually the reference lists of relevant reviews and included studies to capture additional potentially eligible studies.

### Study selection

Two independent investigators (Y.-H.D. and X.T.) removed duplicate records, checked the relevance based on titles and abstracts, and reviewed eventually full-text to determine which studies were eligible for our inclusion criteria after the electronic searches were completed. The following criteria were used to examine the eligibility of published RCTs: (i) patients: adult patients undergoing colorectal resection, regardless of surgical approach (open, laparoscopic, hybrid, hand assisted); (ii) intervention: use of CG, irrespective of category (sugarless or sugared) and method of usage (the quantity, frequency, and duration of CG); (iii) comparison: SPCPs; and (iv) reporting one or more of the outcomes described below. We excluded studies without outcomes of interest. Experimental trials and non-original articles including comment, editorial, and letter to the editor were also excluded from our study. Any divergences on eligibility of studies were resolved by consulting a third investigator (G.-M.S.).

### Data extraction

Two investigators (Y.-H.D. and X.T.) extracted the following information from each study independently using a standardized Excel (Microsoft Corporation) file: first author, year of publication, number of patients, surgical approach, details of chewing gum and SPCPs, demographic characteristics, and outcomes. When we found duplicate reports of the same study in preliminary abstracts and articles, we analyzed data from the most complete dataset. Discrepancies were resolved by discussion between the two investigators.

### Outcome variables and definitions

The primary outcomes were time to first flatus (defined by authors of individual trials), time to first bowel movement (defined by authors of individual trials), time to first bowel sounds (defined by authors of individual trials), time to first feeding (defined by authors of individual trials), and LOS (defined as the time from admission to surgical care unit to hospital discharge or death). Secondary outcomes included: overall complications, other complications including pulmonary infarction, cholecystitis and delirium, postoperative ileus (defined as lack of passage of flatus or stool and intolerance to oral intake for at least 24 h), nausea, vomiting, abdominal distension, overall infections, wound infection, other infections, bleeding, wound dehiscence, anastomotic leak, complications related to GC, readmission rate, reoperation rate, mortality, tolerability of chewing gum, economic effect, and QoL. All secondary outcomes were defined by authors of individual trials apart from postoperative ileus.

### Assessment of risk of bias

Two investigators (J.-G.Z. and X.T.) adopted the Cochrane risk of bias tool to appraise independently risk of bias [46, 47]. The following each item described in this tool was valued to be ‘low’, ‘unclear’, or ‘high’ based on the matching level between extracted information and assessment criteria: random sequence generation; allocation concealment; blinding of participants and personnel; blinding of outcome assessment; incomplete outcome data; selective reporting; and other bias. As dictated by the Cochrane method, trials were rated to be low risk of bias when all key domains are valued low, while trials were rated to be high risk of bias when any one or more key domains are valued high. Otherwise, trials were rated to be unclear risk of bias. The consensus principle was used to resolve any discrepancies.

### Statistical analysis

We calculated risk ratio (RR) with 95 per cent c.i. and weighted mean difference (WMD) with 95 per cent c.i. to present the dichotomous and continuous data respectively. For continuous data, we also used the median values to perform meta-analysis when mean values were not available [48]. We calculated the Cochrane Q to describe qualitatively the heterogeneity, and I2 was used to quantitate it; a value of over 50 per cent indicated significant heterogeneity [49]. Random-effects model was used to perform all analyses regardless of heterogeneity in the present study. We also conducted sensitive analysis by excluding abstract and study with less than 20 participants per arm respectively for time to first flatus, time to first bowel movement and length of hospital stay. Subgroup analysis was also designed to investigate the effects for different surgical approaches including laparoscopic colectomy, open colectomy, and hybrid method. The funnel plot was drawn to examine the publication bias [50]. P < 0.05 indicated statistical significance. We completed all statistical analyses using RevMan 5.3 (The North Cochrane Centre, Copenhagen, Denmark).

### Trial sequential analysis

The risk of yielding spurious statistical inferences in a cumulative meta-analysis was increasing result from repeated significance testing on sparse and accumulated data [51]. The trial sequential analysis, which is comparable to interim analysis in a single trial, was therefore used to examine whether the accumulative evidence was sufficient power to draw a firm conclusion that is an intervention yielded anticipated effect before required information size was accrued, and thus determine whether the trial should be terminated early [52–55]. Construction of sequential monitory boundary and calculation of required information size were at the core of performing trial sequential analysis [56]. We concluded that further studies were not needed if the trial sequential analysis boundary or the futility zone is crossed [52, 55].

A false positive error of 0.05, a false negative error of 0.20 (corresponding to power of 80 per cent), and an anticipated risk ratio reduction of 20 per cent were used to conduct trial sequential analysis in the present study. For binary outcomes, a control event proportion was obtained from the result of the meta-analysis. For continuous outcomes, the mean difference, variance and diversity were estimated empirically based on all eligible trials entered into software. The analyses were done with trial sequential analysis version 0.9 beta (www.ctu.dk/tsa) [55].

### Quality of evidence

Grading of recommendations assessment, development and evaluation (GRADE) method was used to rate the evidence in order to facilitate decision-making [57]. In this method, the evidence from RCTs was firstly established to has high quality and five down-grading factors including risk of bias, imprecision, indirectness, inconsistency, and publication bias can reduce the level to moderate, low and very low [57, 58].

## CONCLUSIONS

In patients undergoing colorectal surgery, implementation of CG have the potential to enhance resumption of gastrointestinal function through decreased the time to first flatus, bowel movement, and bowel sounds, and shorten LOS. However, whether CG may reduce risk of postoperative ileus would require to further study because insufficient evidences were accrued and objective endpoints such as gastrin should be applied. Because sufficient information sizes have confirmed effect of CG on time to first flatus, time to first bowel movement, time to first bowel sounds, and LOS, and thus more studies should be planned to investigate the safety of CG. Furthermore, further studies focusing on optimal quantity, frequency, and duration of consuming CG should also be planned.

## SUPPLEMENTARY MATERIALS




