# Supplementary material for: Meta-analysis comparing chewing gum versus standard postoperative care after colorectal resection
Source: Oncotarget. 2016 Aug 31;7(43):70066–79. doi: 10.18632/oncotarget.11735 (PMC5342535; doi:10.18632/oncotarget.11735)
Supplement: Supplementary file 1 [file oncotarget-07-70066-s001.pdf]

Meta-analysis comparing chewing gum versus standard postoperative care after colorectal resection

	Gum-chewing			Control				Mean Difference
Study or Subgroup	Mean	SD	Total	Mean	SD	Total	Weight	IV, Random, 95% CI
**2.1.1 Excluding abstract**								
Asao 2002	50.4	12	10	76.8	21.6	9	3.0%	-26.40 [-42.35, -10.45]
Atkinson 2016	168	106.67	199	168	106.67	203	2.2%	0.00 [-20.86, 20.86]
Bagena-Aponte 2010	36	34.6	16	40.5	3.5	16	2.8%	-4.50 [-21.54, 12.54]
Bonventre 2014	40.25	23.25	25	57	27	25	3.4%	-16.75 [-30.72, -2.78]
Cao 2008	56.8	9.8	58	78.8	11.2	57	6.2%	-22.00 [-25.85, -18.15]
Crainic 2009	80.2	5.5	17	72.5	6.4	23	6.2%	7.70 [4.00, 11.40]
Fan 2009	76.5	4.3	21	80.6	5.4	21	6.3%	-4.10 [-7.05, -1.15]
Forrester 2014	71.64	78.42	13	82.11	52.31	17	0.5%	-10.47 [-59.82, 38.88]
Hirayama 2006	55.3	15.1	10	90	18	14	3.6%	-34.70 [-47.99, -21.41]
Kobayashi 2015	53	2	21	49	26	22	4.2%	4.00 [-6.90, 14.90]
Li 2012	21.39	10.26	38	36.68	11.27	35	5.9%	-15.29 [-20.25, -10.33]
Lim 2013	42.75	3.92	77	50.97	3.79	80	6.6%	-8.22 [-9.43, -7.01]
Matros 2006	66	22	22	72	28.3	21	3.2%	-6.00 [-21.20, 9.20]
Quah 2006	57.6	24	19	64.8	24	19	3.1%	-7.20 [-22.46, 8.06]
Schuster 2006	65.4	14.8	17	80.2	19.1	17	4.1%	-14.80 [-26.29, -3.31]
Tian 2013	62.52	19.99	50	70.68	19.34	50	5.2%	-8.16 [-15.87, -0.45]
Topcu 2016	51.07	19.63	30	87.83	25.89	30	4.0%	-36.76 [-48.39, -25.13]
Wang 2011	50.38	12.64	78	78.83	13.82	77	6.1%	-28.45 [-32.62, -24.28]
Wang 2016	15.34	3.23	76	30.65	4.83	34	6.5%	-15.31 [-17.09, -13.53]
Zaghiyan 2013	48.6	33.4	54	47.4	29.4	60	4.0%	1.20 [-10.41, 12.81]
Zhang 2015	18.86	4.48	52	26.03	5.11	52	6.5%	-7.17 [-9.02, -5.32]
Zhong 2009	67.7	8.7	60	82	7.6	60	6.3%	-14.30 [-17.22, -11.38]
**Subtotal (95% CI)**			**963**			**942**	**100.0%**	**-12.19 [-15.93, -8.45]**
Heterogeneity: Tau² = 55.10; Chi² = 330.86, df = 21 (P < 0.00001); I² = 94%								
Test for overall effect: Z = 6.39 (P < 0.00001)								
**2.1.2 Excluding small study**								
Atkinson 2016	168	106.67	199	168	106.67	203	2.2%	0.00 [-20.86, 20.86]
Bonventre 2014	40.25	23.25	25	57	27	25	3.6%	-16.75 [-30.72, -2.78]
Cao 2008	56.8	9.8	58	78.8	11.2	57	7.6%	-22.00 [-25.85, -18.15]
Fan 2009	76.5	4.3	21	80.6	5.4	21	7.9%	-4.10 [-7.05, -1.15]
Kobayashi 2015	53	2	21	49	26	22	4.7%	4.00 [-6.90, 14.90]
Li 2012	21.39	10.26	38	36.68	11.27	35	7.2%	-15.29 [-20.25, -10.33]
Lim 2013	42.75	3.92	77	50.97	3.79	80	8.2%	-8.22 [-9.43, -7.01]
Matros 2006	66	22	22	72	28.3	21	3.3%	-6.00 [-21.20, 9.20]
McCormick 2005	57.6	24	62	62.4	33.6	40	4.3%	-4.80 [-16.80, 7.20]
Tian 2013	62.52	19.99	50	70.68	19.34	50	6.0%	-8.16 [-15.87, -0.45]
Topcu 2016	51.07	19.63	30	87.83	25.89	30	4.4%	-36.76 [-48.39, -25.13]
Wang 2011	50.38	12.64	78	78.83	13.82	77	7.5%	-28.45 [-32.62, -24.28]
Wang 2016	15.34	3.23	76	30.65	4.83	34	8.2%	-15.31 [-17.09, -13.53]
Watson 2010	47	19.75	26	67	20.4	27	4.7%	-20.00 [-30.81, -9.19]
Zaghiyan 2013	48.6	33.4	54	47.4	29.4	60	4.4%	1.20 [-10.41, 12.81]
Zhang 2015	18.86	4.48	52	26.03	5.11	52	8.1%	-7.17 [-9.02, -5.32]
Zhong 2009	67.7	8.7	60	82	7.6	60	7.9%	-14.30 [-17.22, -11.38]
**Subtotal (95% CI)**			**949**			**894**	**100.0%**	**-12.66 [-16.22, -9.11]**
Heterogeneity: Tau² = 39.53; Chi² = 223.46, df = 16 (P < 0.00001); I² = 93%								
Test for overall effect: Z = 6.98 (P < 0.00001)								

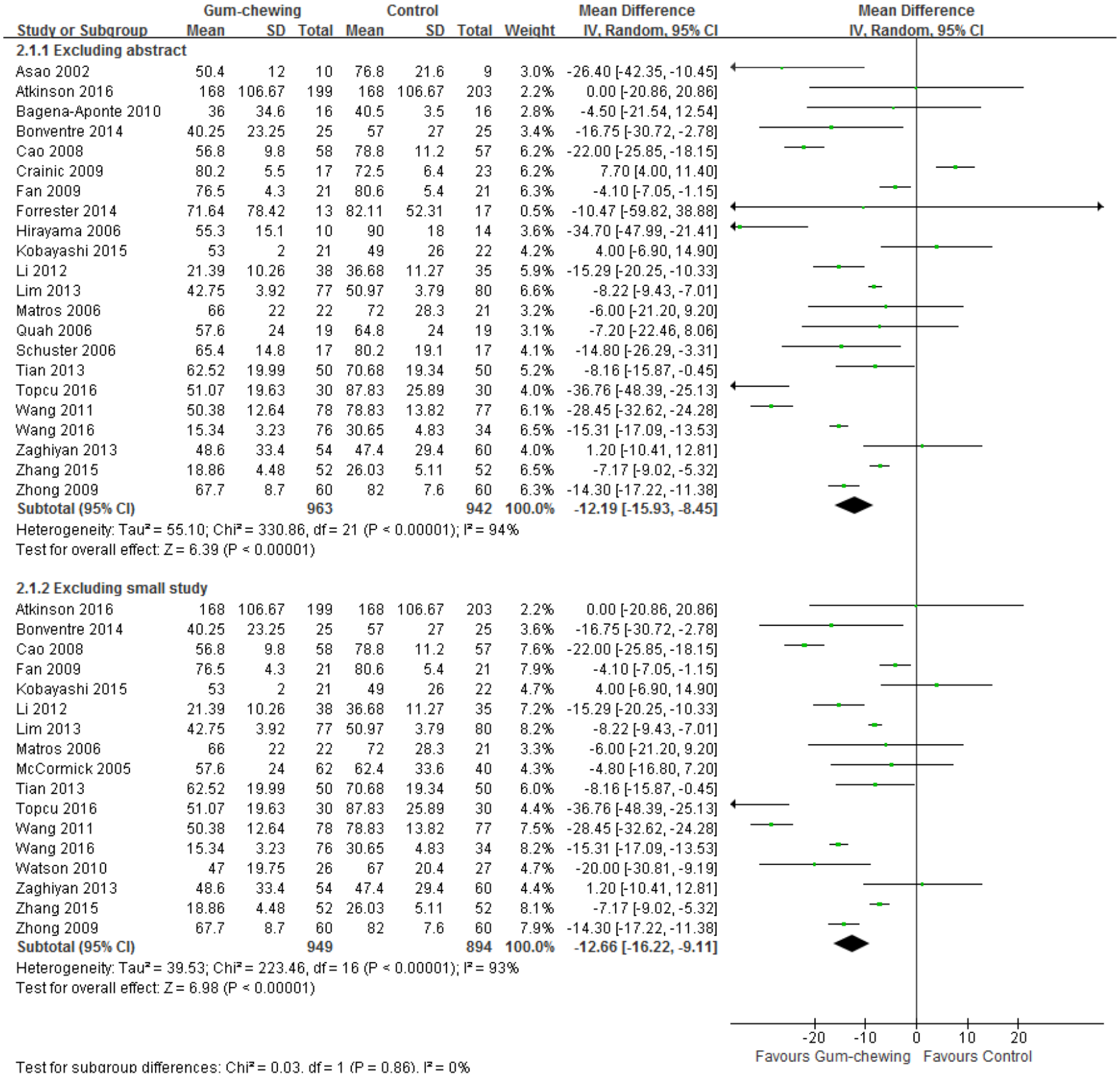

Test for subgroup differences: Chi² = 0.03, df = 1 (P = 0.86), I² = 0%

A. Time to first flatus (hours)

(*Continued*)

Study or Subgroup	Gum-chewing Mean	SD	Total	Control Mean	SD	Total	Weight	Mean Difference IV, Random, 95% CI
**3.1.1 Excluding abstract**								
Asao 2002	74.4	26.4	10	139.2	52.8	9	2.1%	-64.80 [-102.98, -26.62]
Atkinson 2016	48	16	159	72	16	151	7.7%	-24.00 [-27.56, -20.44]
Bagena-Aponte 2010	44.2	17.6	16	55	14.8	16	6.4%	-10.80 [-22.07, 0.47]
Bonventre 2014	99.25	54.25	25	108	48	25	3.2%	-8.75 [-37.14, 19.64]
Crainic 2009	90.9	5.8	19	92.7	9.1	20	7.6%	-1.80 [-6.57, 2.97]
Fan 2009	80.8	5.8	21	85.4	7.8	21	7.6%	-4.60 [-8.76, -0.44]
Forrester 2014	125.06	110.8	9	79.95	36.43	15	0.7%	45.11 [-29.59, 119.81]
Hirayama 2006	84.5	37.8	10	136	56.8	14	2.2%	-51.50 [-89.37, -13.63]
Kobayashi 2015	94	44	21	109	35	22	3.8%	-15.00 [-38.83, 8.83]
Li 2012	48.57	9.26	38	57.39	6.55	35	7.7%	-8.82 [-12.48, -5.16]
Lim 2013	89.64	5.94	77	98.61	7.06	80	7.8%	-8.97 [-11.01, -6.93]
Matros 2006	86	30	22	93	36	21	4.6%	-7.00 [-26.86, 12.86]
Quah 2006	76.8	36	19	93.6	36	19	4.0%	-16.80 [-39.69, 6.09]
Schuster 2006	63.2	5.4	17	89.4	24	17	6.3%	-26.20 [-37.89, -14.51]
Tian 2013	84.2	14.25	50	91.7	19.39	50	7.3%	-7.50 [-14.17, -0.83]
Topcu 2016	76.47	14.83	78	117.87	15.65	77	7.6%	-41.40 [-46.20, -36.60]
Wang 2011	56.9	37.8	54	63.2	41.9	60	5.6%	-6.30 [-20.93, 8.33]
Zaghiyan 2013	41.4	2.8	60	67.6	4.1	60	7.9%	-26.20 [-27.46, -24.94]
Zhong 2009	0	0	0	0	0	0		Not estimable
**Subtotal (95% CI)**			**705**			**712**	**100.0%**	**-16.17 [-22.68, -9.65]**
Heterogeneity: $Tau^2 = 140.03$; $Chi^2 = 465.24$, df = 17 ($P < 0.00001$); $I^2 = 96\%$								
Test for overall effect: Z = 4.86 ($P < 0.00001$)								
**3.1.2 Excluding small study**								
Atkinson 2016	48	16	159	72	16	151	9.3%	-24.00 [-27.56, -20.44]
Bonventre 2014	99.25	54.25	25	108	48	25	3.7%	-8.75 [-37.14, 19.64]
Fan 2009	80.8	5.8	21	85.4	7.8	21	9.2%	-4.60 [-8.76, -0.44]
Kobayashi 2015	94	44	21	109	35	22	4.5%	-15.00 [-38.83, 8.83]
Li 2012	48.57	9.26	38	57.39	6.55	35	9.3%	-8.82 [-12.48, -5.16]
Lim 2013	89.64	5.94	77	98.61	7.06	80	9.4%	-8.97 [-11.01, -6.93]
Matros 2006	86	30	22	93	36	21	5.4%	-7.00 [-26.86, 12.86]
McCormick 2005	69.6	26.4	62	84	31.2	40	7.5%	-14.40 [-26.09, -2.71]
Tian 2013	84.2	14.25	50	91.7	19.39	50	8.7%	-7.50 [-14.17, -0.83]
Wang 2011	76.47	14.83	78	117.87	15.65	77	9.1%	-41.40 [-46.20, -36.60]
Watson 2010	74	13	26	112.5	24.25	27	7.8%	-38.50 [-48.92, -28.08]
Zaghiyan 2013	56.9	37.8	54	63.2	41.9	60	6.7%	-6.30 [-20.93, 8.33]
Zhong 2009	41.4	2.8	60	67.6	4.1	60	9.5%	-26.20 [-27.46, -24.94]
**Subtotal (95% CI)**			**693**			**669**	**100.0%**	**-17.10 [-24.06, -10.13]**
Heterogeneity: $Tau^2 = 133.03$; $Chi^2 = 407.24$, df = 12 ($P < 0.00001$); $I^2 = 97\%$								
Test for overall effect: Z = 4.81 ($P < 0.00001$)								

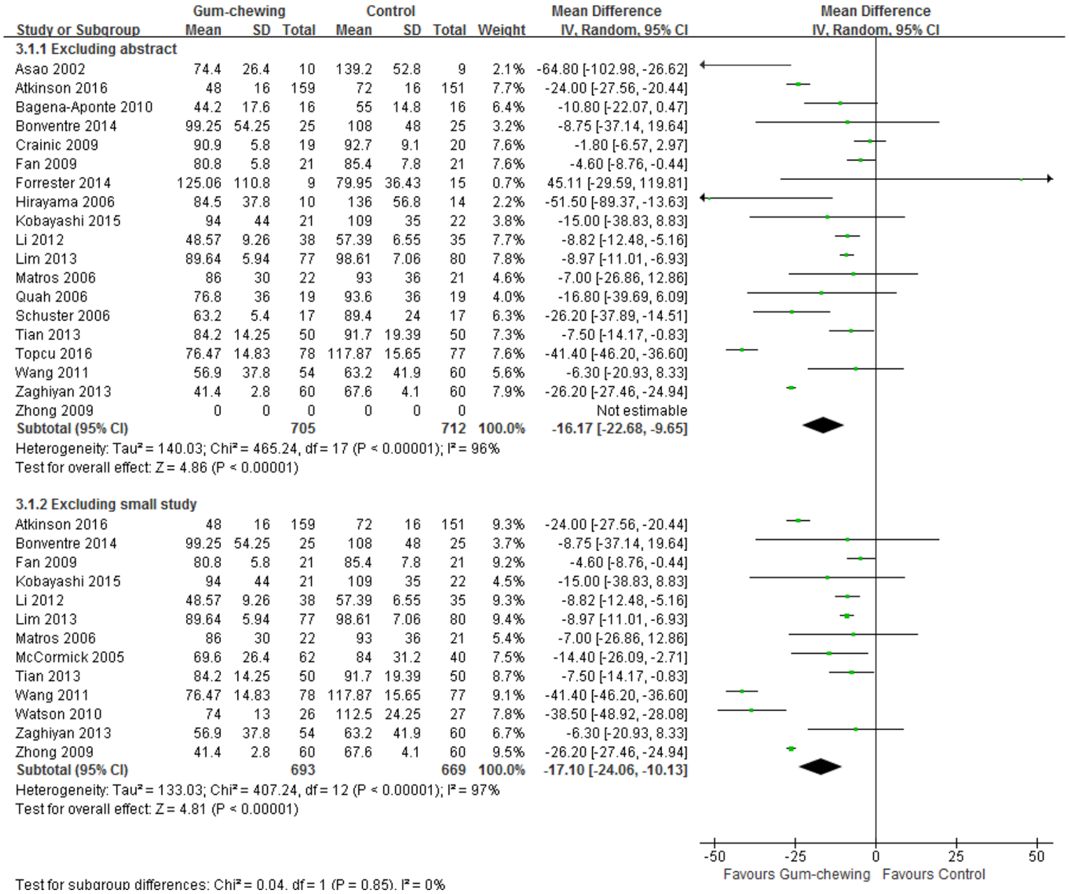

Test for subgroup differences: $Chi^2 = 0.04$, df = 1 ($P = 0.85$), $I^2 = 0\%$

B. time to first bowel movement (hours)

(*Continued*)

	Gum-chewing			Control				Mean Difference
Study or Subgroup	Mean	SD	Total	Mean	SD	Total	Weight	IV, Random, 95% CI
**4.1.1 Excluding abstract**								
Asao 2002	13.5	3	10	14.5	6.1	9	2.2%	-1.00 [-5.40, 3.40]
Atkinson 2016	7	61.3	198	7	38	202	0.5%	0.00 [-10.02, 10.02]
Bagena-Aponte 2010	4.25	12.7	16	6.25	15.5	16	0.5%	-2.00 [-11.82, 7.82]
Bonventre 2014	6.75	2.25	25	7	2.5	25	8.4%	-0.25 [-1.57, 1.07]
Fan 2009	13.6	1.3	21	14.2	1.5	21	10.1%	-0.60 [-1.45, 0.25]
Forrester 2014	6.31	5.47	13	6.26	5.83	18	2.6%	0.05 [-3.96, 4.06]
Heijkant 2015	9.5	4.9	52	14	14.5	60	2.7%	-4.50 [-8.40, -0.60]
Kobayashi 2015	26.3	12.2	21	21.2	17.8	22	0.6%	5.10 [-3.99, 14.19]
Li 2012	6.24	1.78	38	7.31	1.07	35	10.6%	-1.07 [-1.74, -0.40]
Lim 2013	9.97	12.7	77	12.03	15.5	80	2.2%	-2.06 [-6.49, 2.37]
Matros 2006	5.17	1.58	22	5.71	3	21	8.0%	-0.54 [-1.98, 0.90]
Quah 2006	9.4	2.5	19	11.1	7.3	19	3.2%	-1.70 [-5.17, 1.77]
Schuster 2006	4.3	0.43	17	6.8	1.38	17	10.6%	-2.50 [-3.19, -1.81]
Topcu 2016	7.63	1.47	30	9.47	2.67	30	9.2%	-1.84 [-2.93, -0.75]
Wang 2011	8.35	3.44	78	11.73	4.26	77	8.8%	-3.38 [-4.60, -2.16]
Zaghiyan 2013	4.7	0.7	54	4.5	1.9	60	11.0%	0.20 [-0.32, 0.72]
Zhong 2009	11.4	2.8	60	11.6	3.8	60	8.9%	-0.20 [-1.39, 0.99]
**Subtotal (95% CI)**			**751**			**772**	**100.0%**	**-1.18 [-1.91, -0.46]**
Heterogeneity: Tau² = 1.18; Chi² = 65.74, df = 16 (P < 0.00001); I² = 76%								
Test for overall effect: Z = 3.20 (P = 0.001)								
**4.1.2 Excluding small study**								
Atkinson 2016	7	61.3	198	7	38	202	0.3%	0.00 [-10.02, 10.02]
Bonventre 2014	6.75	2.25	25	7	2.5	25	8.1%	-0.25 [-1.57, 1.07]
Fan 2009	13.6	1.3	21	14.2	1.5	21	10.6%	-0.60 [-1.45, 0.25]
Heijkant 2015	9.5	4.9	52	14	14.5	60	2.0%	-4.50 [-8.40, -0.60]
Kobayashi 2015	26.3	12.2	21	21.2	17.8	22	0.4%	5.10 [-3.99, 14.19]
Li 2012	6.24	1.78	38	7.31	1.07	35	11.5%	-1.07 [-1.74, -0.40]
Lim 2013	9.97	12.7	77	12.03	15.5	80	1.6%	-2.06 [-6.49, 2.37]
Matros 2006	5.17	1.58	22	5.71	3	21	7.5%	-0.54 [-1.98, 0.90]
McCormick 2005	4.4	1.5	62	5.2	2	40	11.2%	-0.80 [-1.52, -0.08]
Topcu 2016	7.63	1.47	30	9.47	2.67	30	9.3%	-1.84 [-2.93, -0.75]
Wang 2011	8.35	3.44	78	11.73	4.26	77	8.6%	-3.38 [-4.60, -2.16]
Watson 2010	5.42	2.37	26	6.13	2.77	27	7.8%	-0.71 [-2.10, 0.68]
Zaghiyan 2013	4.7	0.7	54	4.5	1.9	60	12.2%	0.20 [-0.32, 0.72]
Zhong 2009	11.4	2.8	60	11.6	3.8	60	8.7%	-0.20 [-1.39, 0.99]
**Subtotal (95% CI)**			**764**			**760**	**100.0%**	**-0.95 [-1.55, -0.35]**
Heterogeneity: Tau² = 0.69; Chi² = 42.49, df = 13 (P < 0.0001); I² = 69%								
Test for overall effect: Z = 3.12 (P = 0.002)								

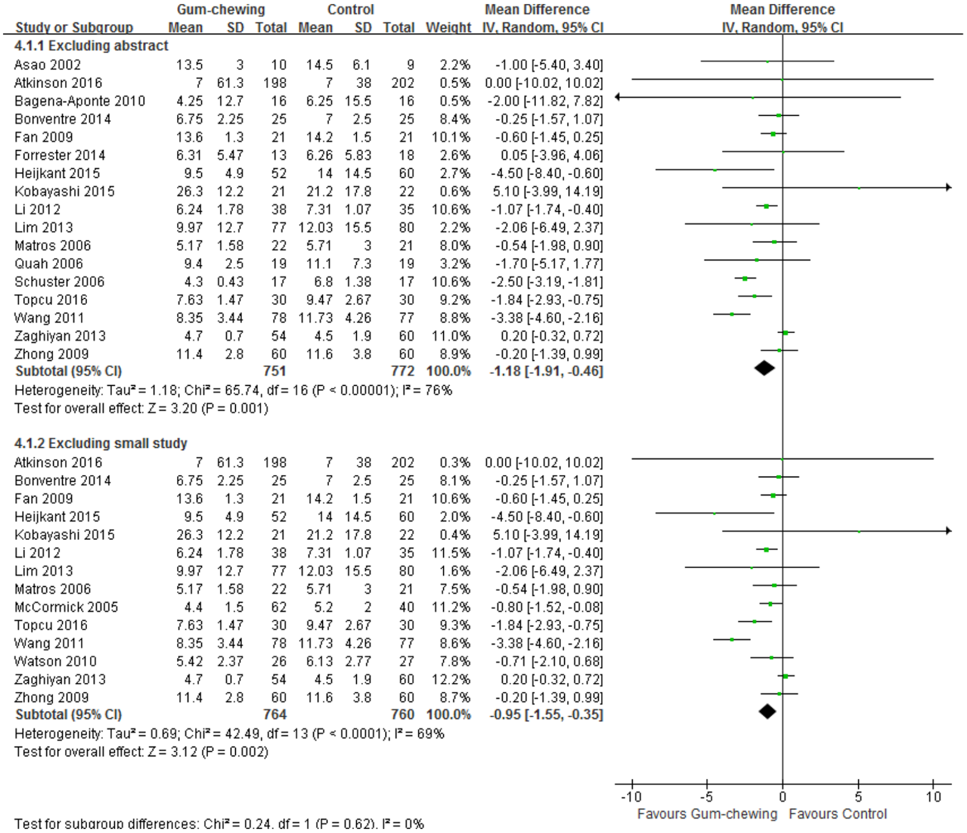

Test for subgroup differences: Chi² = 0.24, df = 1 (P = 0.62), I² = 0%

C. Length of hospital stay (days)

Supplementary Figure S1: Sensitive analysis for testing the robust of summarized results by excluding abstract with essential data and study with less than 20 patients per arm: time to first flatus A., time to first bowel movement **B.**, and length of hospital stay **C.** The green square represents point estimate of each trial, and its bigger size is correspondence to higher weight. The short horizontal line represents the confidence interval of each trial, and the shorter length is correspondence to the more precision of estimating effect size. The black diamond represents the summarized effect size, and the horizontal diagonal is correspondence to confidence interval.

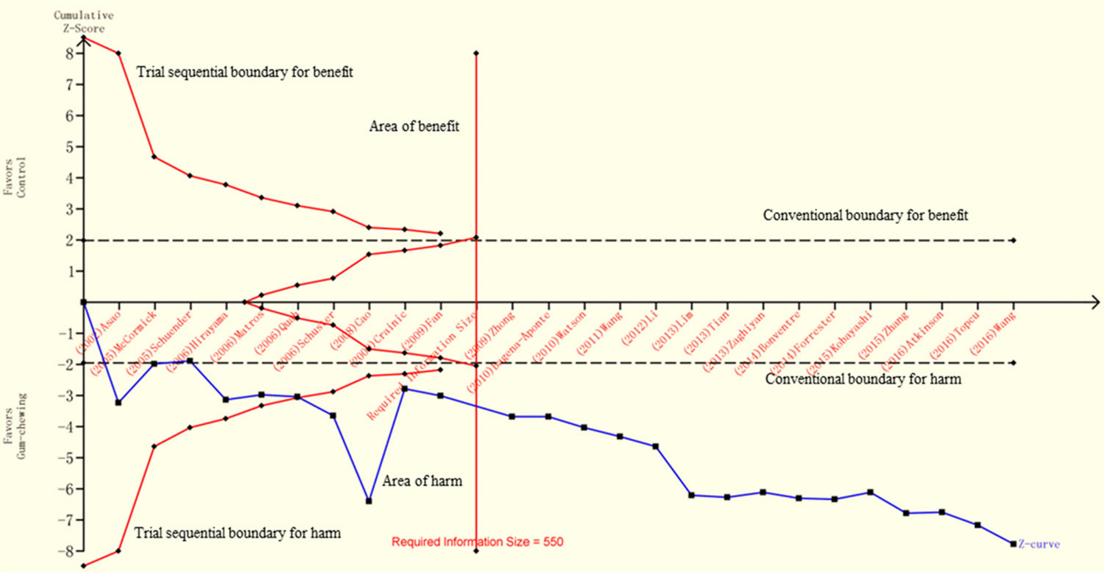

A. Trial sequential analysis of time to first flatus suggesting that the accumulative Z-curve crossed the trial sequential monitoring boundary for harm and surpassed required information size (RIS). The trial sequential analysis showed that 2096 participants were accrued (RIS = 550). The RIS was calculated using α = 0.05 (two-sided), β = 0.20 (power 0.80), a variance of 146.7, a diversity of 94 %, and a mean difference of -11.9 hours. The trial sequential analysis confirmed the evidence which gum-chewing significantly reduced the time to first flatus. The variance and mean difference were estimated empirically.

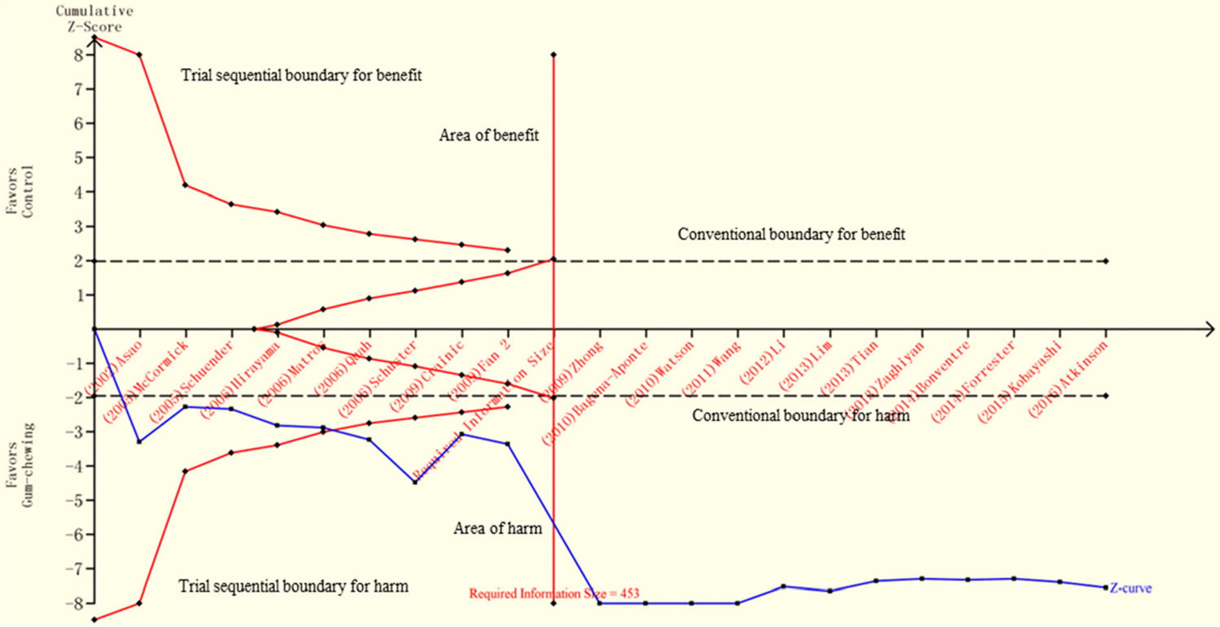

B. Trial sequential analysis of time to first bowel movement suggesting that the accumulative Z-curve crossed the trial sequential monitoring boundary for harm and surpassed required information size (RIS). The trial sequential analysis showed that 1608 participants were accrued (RIS = 453). The RIS was calculated using α = 0.05 (two-sided), β = 0.20 (power 0.80), a variance of 168.6, a diversity of 96 %, and a mean difference of -17.38 hours. The trial sequential analysis confirmed the evidence which gum-chewing significantly shortened the time to first bowel movement. The variance and mean difference were estimated empirically.

(Continued)

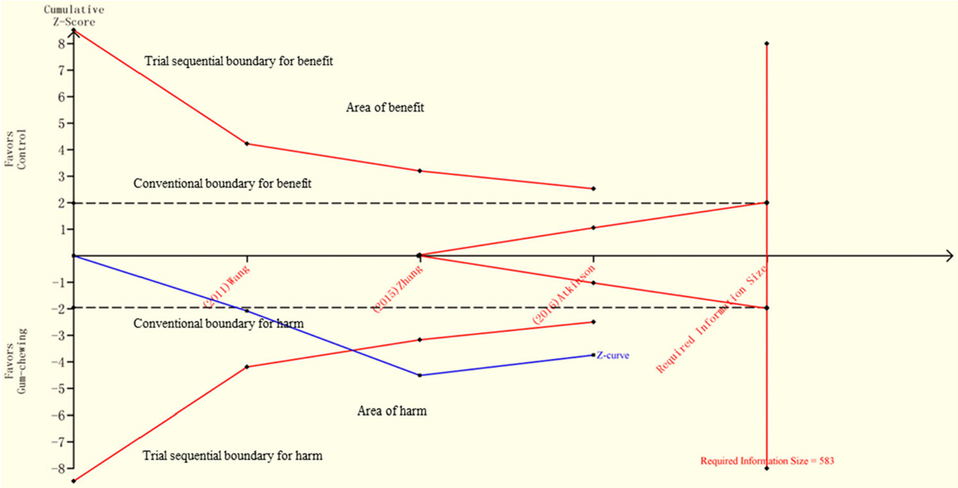

C. Trial sequential analysis of time to first bowel sounds suggesting that the accumulative Z-curve crossed the trial sequential monitoring boundary for harm after tenth trial. The trial sequential analysis showed that 505 participants were accrued (required information size = 583). The RIS was calculated using α = 0.05 (two-sided), β = 0.20 (power 0.80), a variance of 261.7, a diversity of 30 %, and a mean difference of -5.18 hours. The trial sequential analysis confirmed the evidence which gum-chewing significantly reduced the time to first bowel sounds. The variance and mean difference were estimated empirically. After entered data of all four trials into trial sequential analysis software, the system notification indicated that required information size is not renderable due to first statistical fraction exceeds 100% of required information size. We therefor excluded the trial by Wang (2016) to re-perform trial sequential analysis.

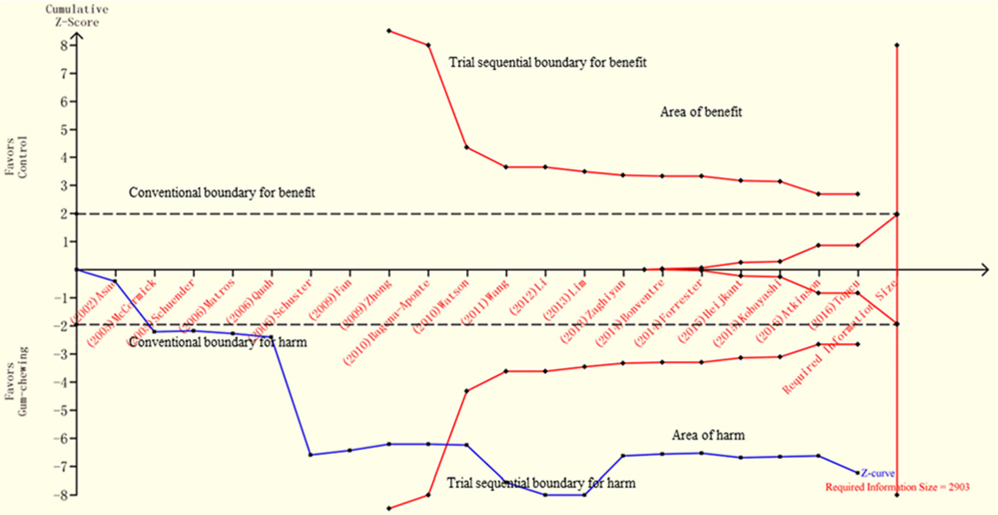

D. Trial sequential analysis of time to length of hospital stay suggesting that the accumulative Z-curve crossed the trial sequential monitoring boundary for harm after tenth trial. The trial sequential analysis showed that 1714 participants were accrued (required information size = 2903). The RIS was calculated using α = 0.05 (two-sided), β = 0.20 (power 0.80), a variance of 14.33, a diversity of 82 %, and a mean difference of -0.94 hours. The trial sequential analysis confirmed the evidence which gum-chewing significantly reduced the length of hospital stays. The variance and mean difference were estimated empirically.

Supplementary Figure S2: Trial sequential analysis of continuous outcomes: time to first flatus A., time to first bowel movement **B.**, time to first bowel sounds **C.** and length of hospital stays **D.** Trial sequential analysis is a technique to confirm whether cumulative information size can obtain anticipant effects before the required information size was accrued. For meta-analysis of continuous data, a diversity-adjusted required information size and the trial sequential boundaries can be calculated and constructed respectively using a reasonable α, β, mean difference, variance, and diversity (D^2). And then statistical inferences could be drawn regarding the relationship between cumulative meta-analysis (Z-curve) and required information size: if the Z-curve crossed the trial sequential monitoring boundary or the futility zone before the required information size is reached, firm evidence may perhaps be established and further trials should not be warranted; in contrast, if Z-curve did not cross the boundary, there is insufficient evidence to detect or reject a certain effect of the intervention and more trials are still needed.

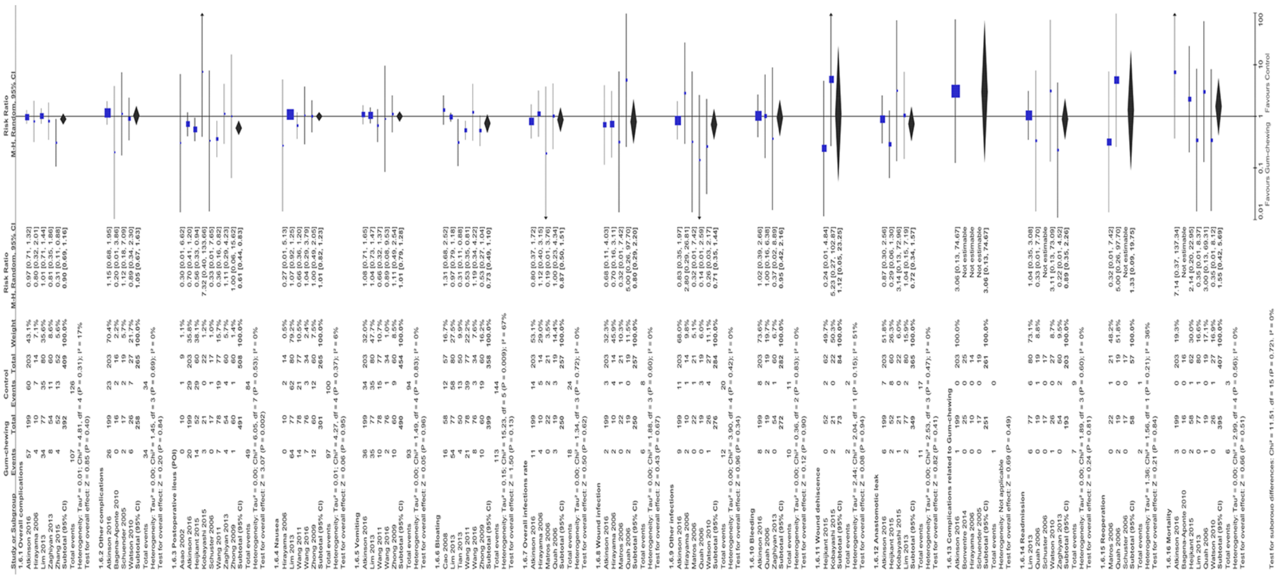

Supplementary Figure S3: Forest plot of secondary outcomes. The summary effect estimate (risk ratio, RR) for individual randomized controlled trials (RCTs) are indicated by green rectangles (the size of the rectangle is proportional to the study weight), with the black horizontal lines representing 95 per cent c.i. The overall summary effect estimate and 95 per cent c.i. are indicated by the black diamond below. M-H = Mantel-Haenszel.

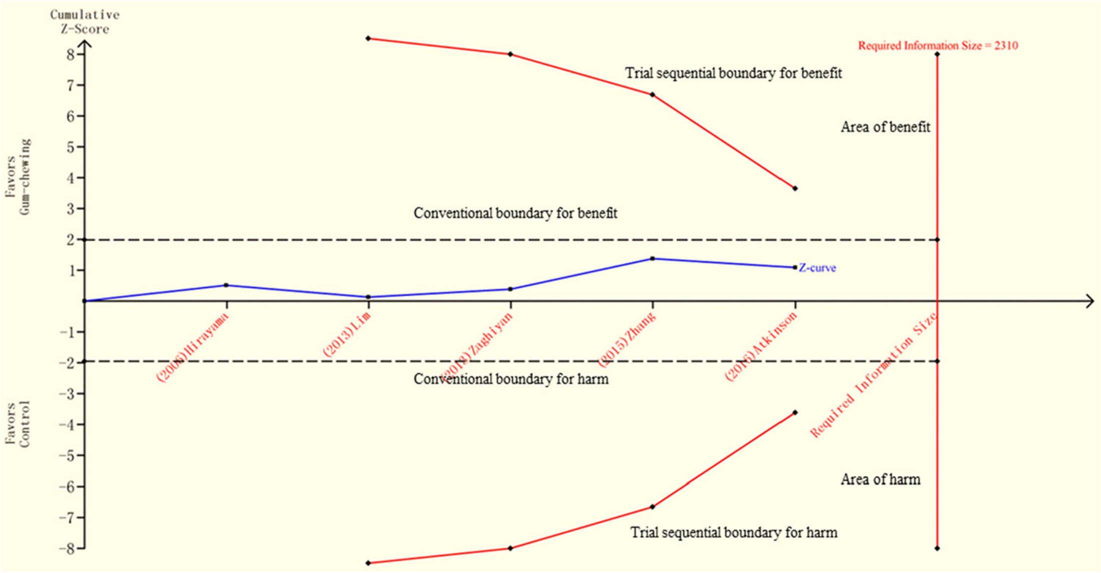

A. Trial sequential analysis of overall infection suggesting that 233 participants were accrued (required information size = 2310). The required information size was calculated using α = 0.05 (two-sided), β = 0.20 (power 0.80), a relative risk reduction of 20 %, a diversity of 28 %, and a control event proportion of 30.81 % obtained from the results of the meta-analysis. The trial sequential analysis indicated that the cumulative Z-curve did not cross the conventional boundary for benefit. So we concluded that there is no sufficient evidence to detect the difference between the gum-chewing and control groups.

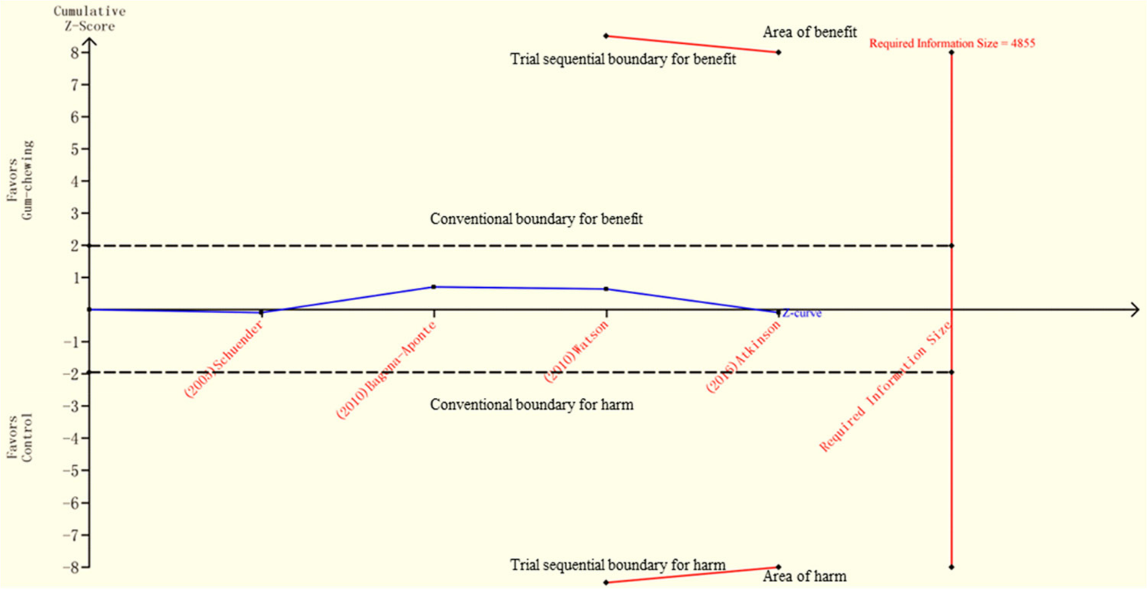

B. Trial sequential analysis of other infections suggesting that 523 participants were accrued (required information size = 4855). The required information size was calculated using α = 0.05 (two-sided), β = 0.20 (power 0.80), a relative risk reduction of 20 %, a diversity of 0 %, and a control event proportion of 12.83 % obtained from the results of the meta-analysis. The trial sequential analysis indicated that the cumulative Z-curve did not cross the conventional boundary for benefit and did not accrue the required information size. So we concluded that there is no sufficient evidence to detect the difference between the gum-chewing and control groups.

(*Continued*)

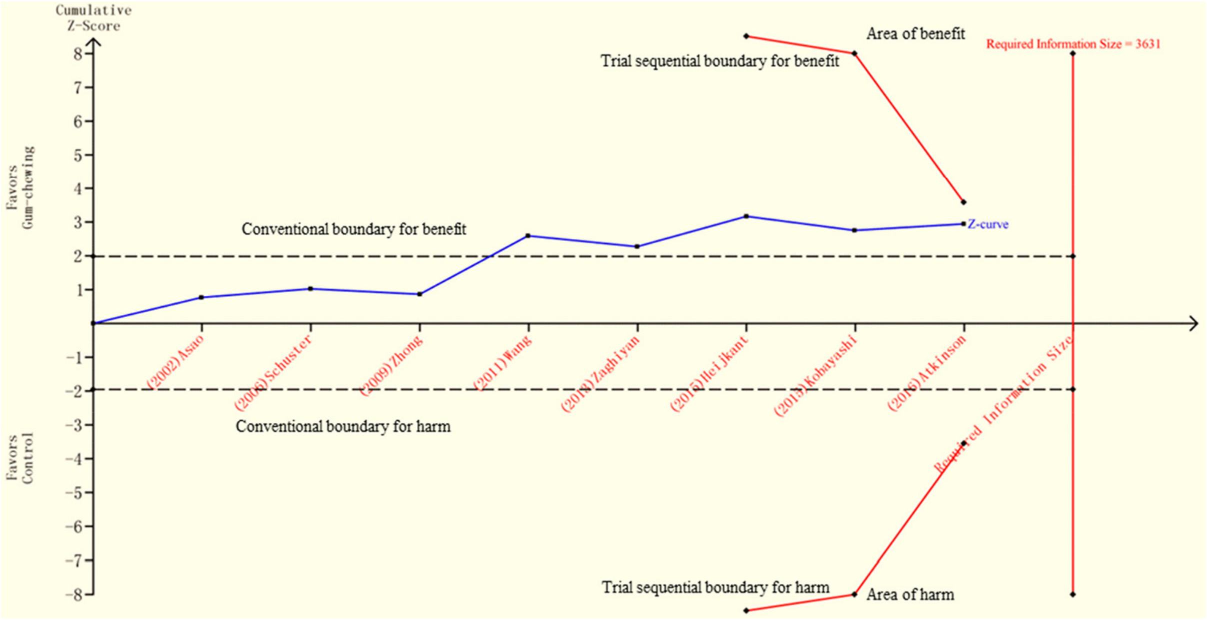

C. Trial sequential analysis of postoperative ileus (POI) suggesting that 999 participants were accrued (required information size = 3631). The required information size was calculated using α = 0.05 (two-sided), β = 0.20 (power 0.80), a relative risk reduction of 20 %, a diversity of 0 %, and a control event proportion of 16.54 % obtained from the results of the meta-analysis. The trial sequential analysis indicated that the cumulative Z-curve crossed the conventional boundary for benefit and did not accrue the required information size. So we concluded that the evidence of detected the difference between the gum-chewing and control groups presented false-positive.

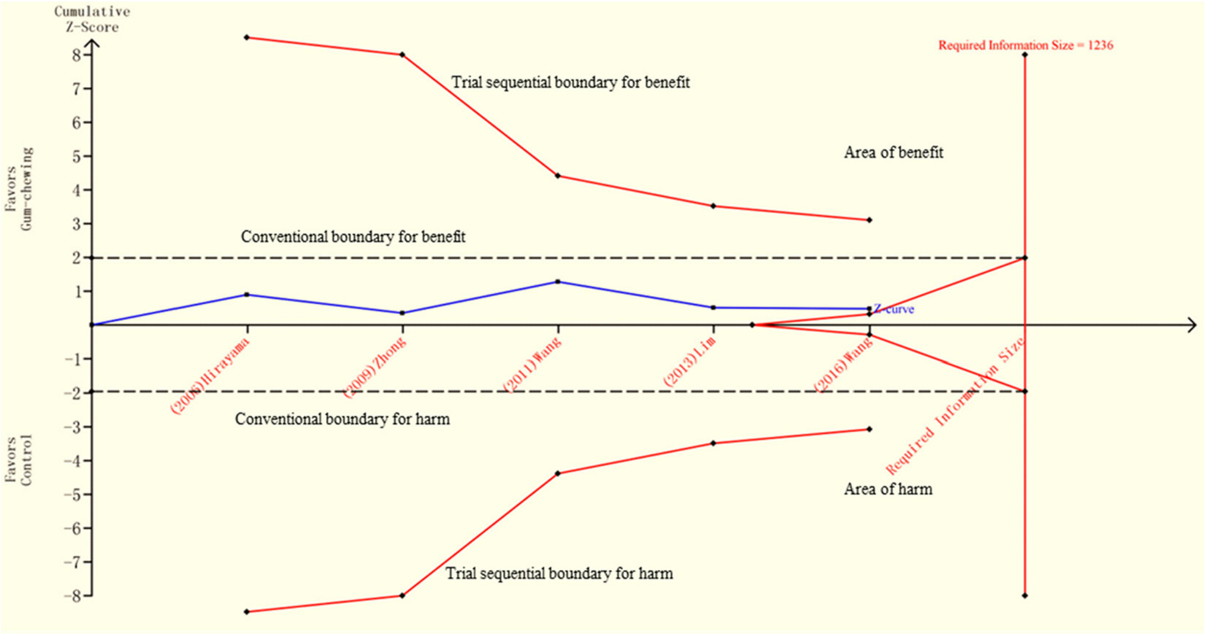

D. Trial sequential analysis of nausea suggesting that 566 participants were accrued (required information size = 1236). The required information size was calculated using α = 0.05 (two-sided), β = 0.20 (power 0.80), a relative risk reduction of 20 %, a diversity of 0 %, and a control event proportion of 37.74 % obtained from the results of the meta-analysis. The trial sequential analysis indicated that the cumulative Z-curve did not cross the conventional boundary for benefit and did not accrue the required information size. So we concluded that there is no sufficient evidence to detect the difference between the gum-chewing and control groups.

(*Continued*)

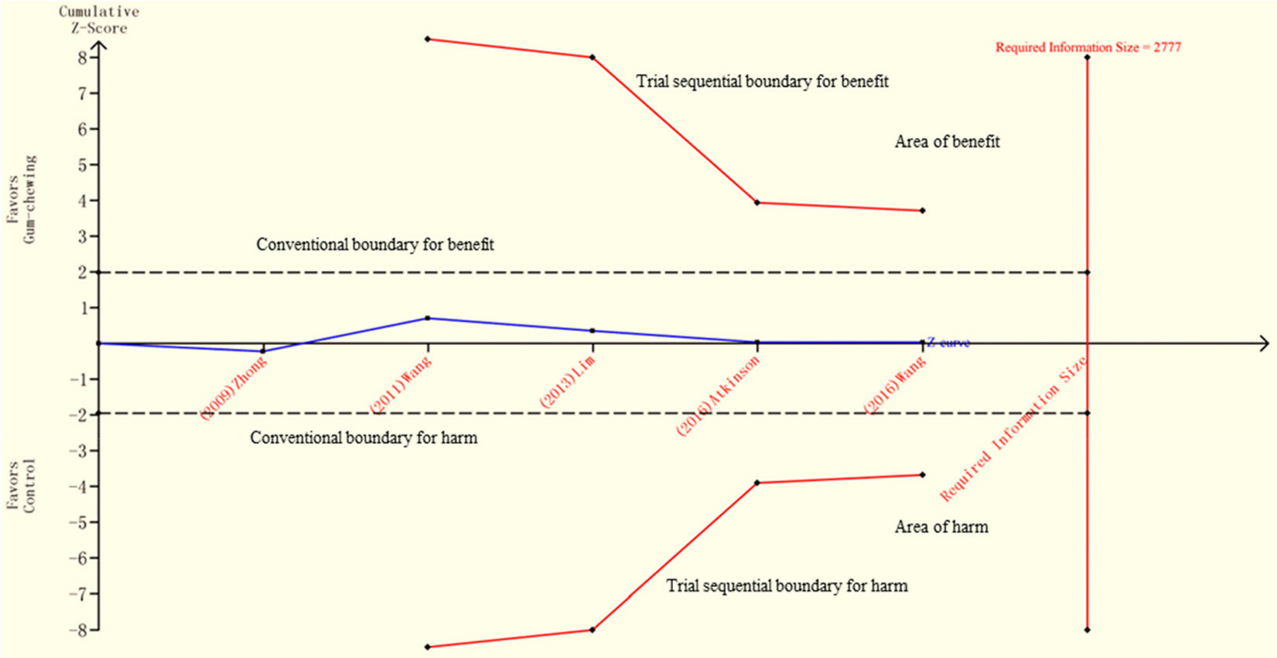

E. Trial sequential analysis of vomiting suggesting that 944 participants were accrued (required information size = 2777). The required information size was calculated using α = 0.05 (two-sided), β = 0.20 (power 0.80), a relative risk reduction of 20 %, a diversity of 0 %, and a control event proportion of 20.7 % obtained from the results of the meta-analysis. The trial sequential analysis indicated that the cumulative Z-curve did not cross the conventional boundary for benefit and did not accrue the required information size. So we concluded that there is no sufficient evidence to detect the difference between the gum-chewing and control groups.

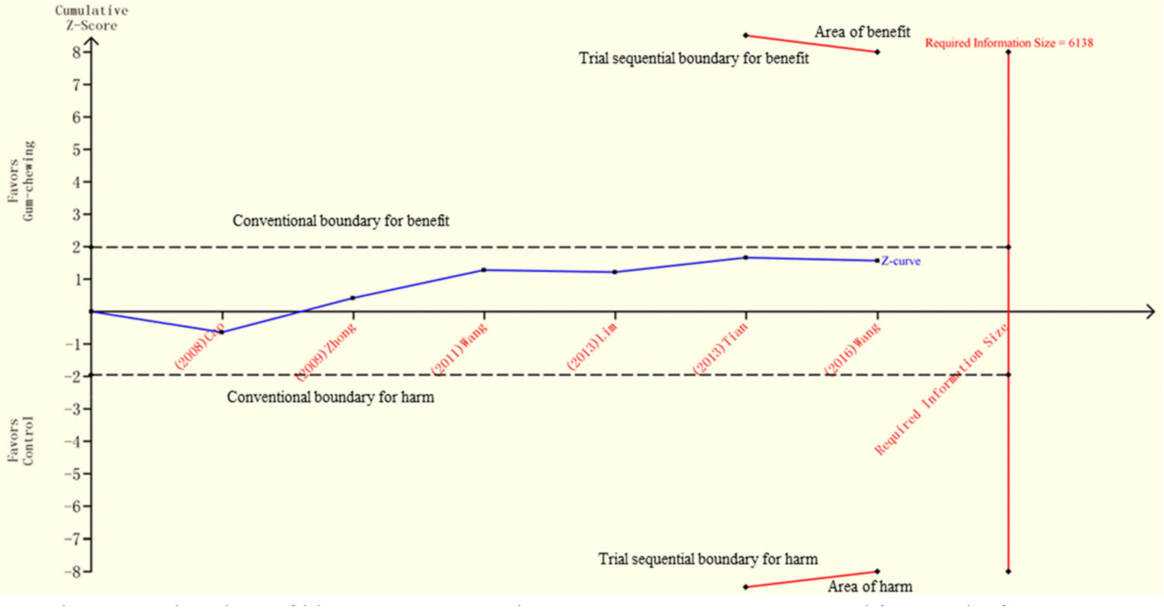

F. Trial sequential analysis of bloating suggesting that 757 participants were accrued (required information size = 6138). The required information size was calculated using α = 0.05 (two-sided), β = 0.20 (power 0.80), a relative risk reduction of 20 %, a diversity of 82 %, and a control event proportion of 40.22 % obtained from the results of the meta-analysis. The trial sequential analysis indicated that the cumulative Z-curve did not cross the conventional boundary for benefit and did not accrue the required information size. So we concluded that there is no sufficient evidence to detect the difference between the gum-chewing and control groups.

(*Continued*)

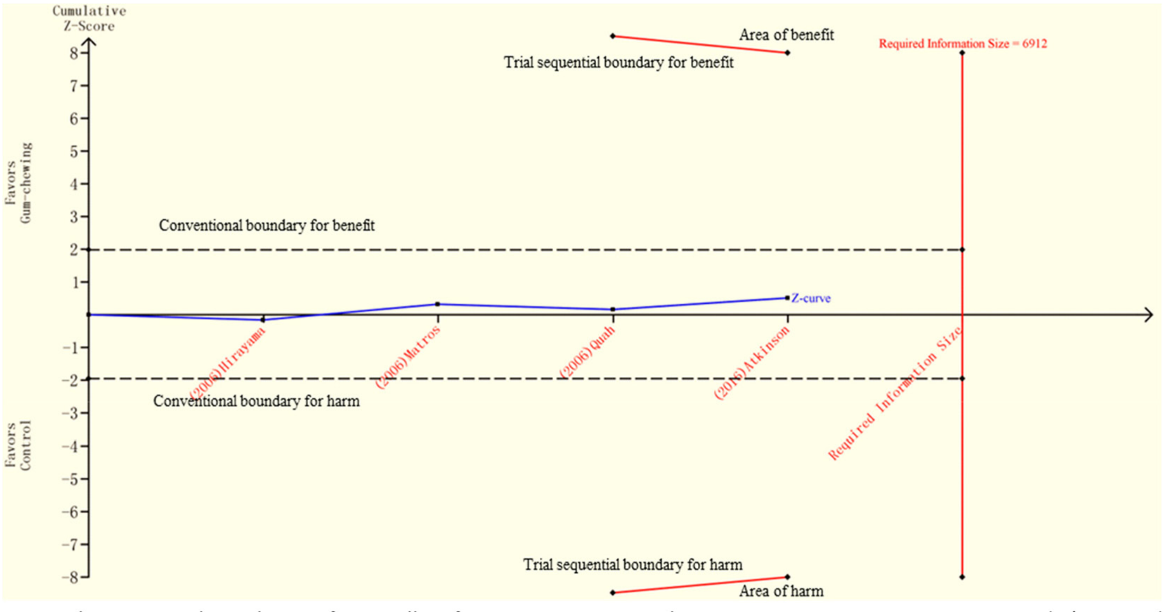

G. Trial sequential analysis of overall infection suggesting that 507 participants were accrued (required information size = 6912). The required information size was calculated using α = 0.05 (two-sided), β = 0.20 (power 0.80), a relative risk reduction of 20 %, a diversity of 0 %, and a control event proportion of 9.34 % obtained from the results of the meta-analysis. The trial sequential analysis exhibited that the cumulative Z-curve did not cross the conventional boundary for benefit and did not accrue the required information size. So we concluded that there is no sufficient evidence to detect the difference between the gum-chewing and control groups.

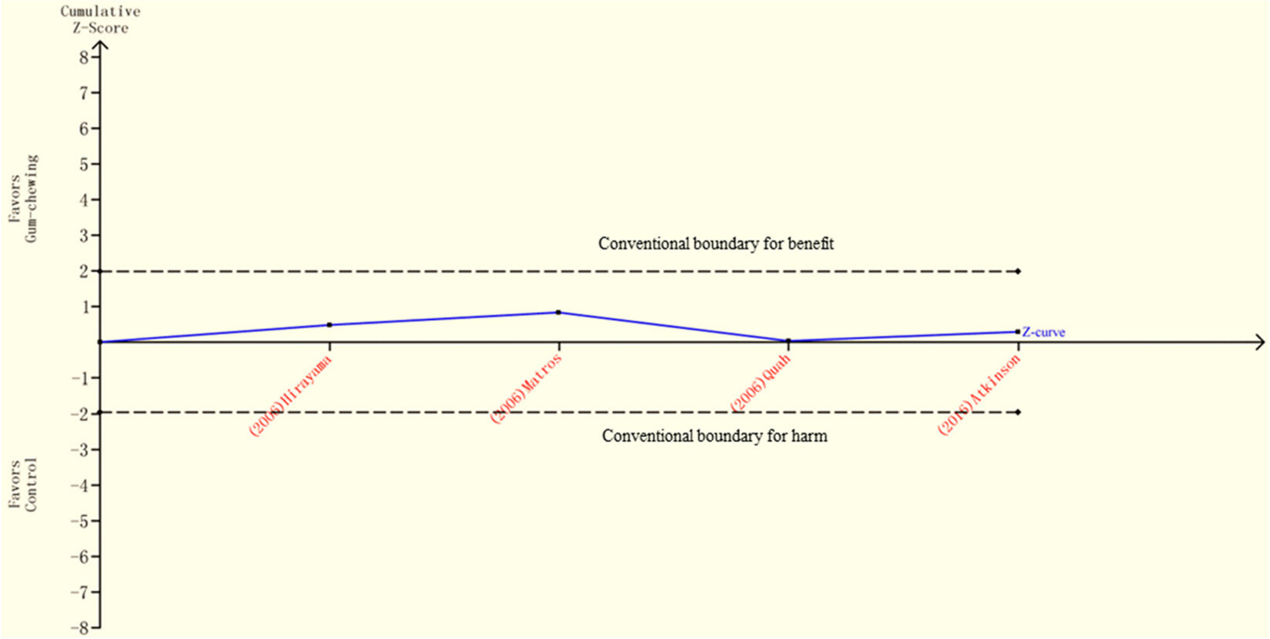

H. Trial sequential analysis of wound infection suggesting that 507 participants were accrued (required information size = 22229). The required information size was calculated using α = 0.05 (two-sided), β = 0.20 (power 0.80), a relative risk reduction of 20 %, a diversity of 0 %, and a control event proportion of 3.11 % obtained from the results of the meta-analysis. The trial sequential analysis exhibited that the cumulative Z-curve did not cross the conventional boundary for benefit and did not accrue the required information size. The trial sequential monitoring boundaries were ignored due to only 2.28 % of required sample size was reached in the trials of the meta-analysis. So we concluded that there is no sufficient evidence to detect the difference between the gum-chewing and control groups.

(*Continued*)

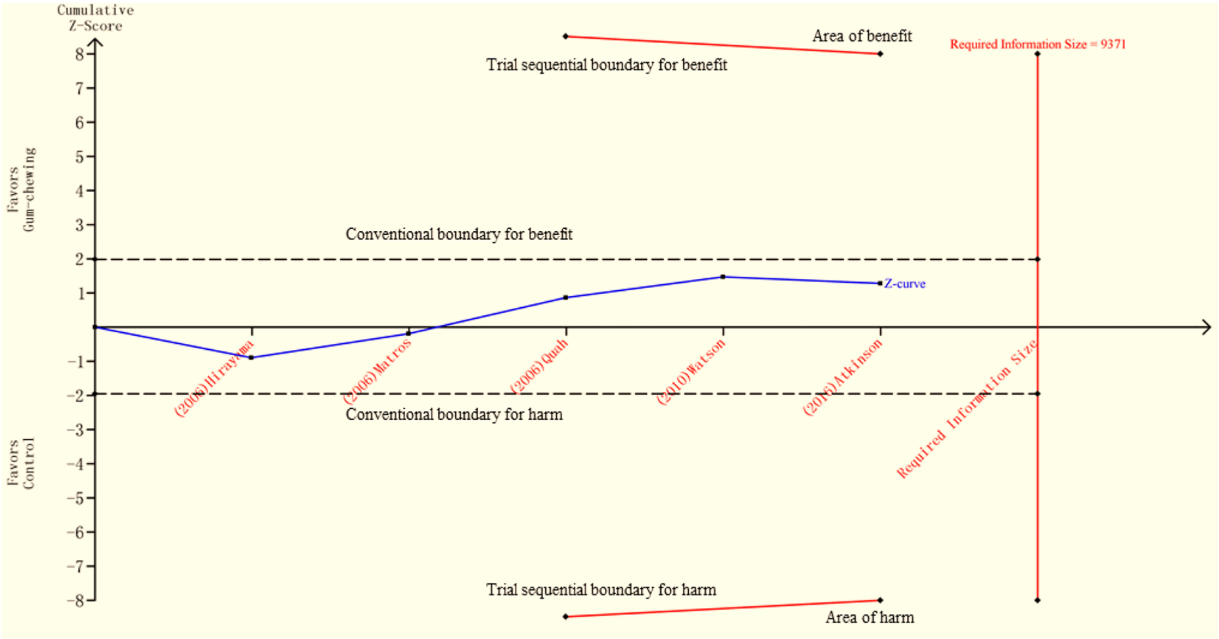

I. Trial sequential analysis of other infections suggesting that 560 participants were accrued (required information size = 9371). The required information size was calculated using α = 0.05 (two-sided), β = 0.20 (power 0.80), a relative risk reduction of 20 %, a diversity of 0 %, and a control event proportion of 7.04 % obtained from the results of the meta-analysis. The trial sequential analysis exhibited that the cumulative Z-curve did not cross the conventional boundary for benefit and did not accrue the required information size. So we concluded that there is no sufficient evidence to detect the difference between the gum-chewing and control groups.

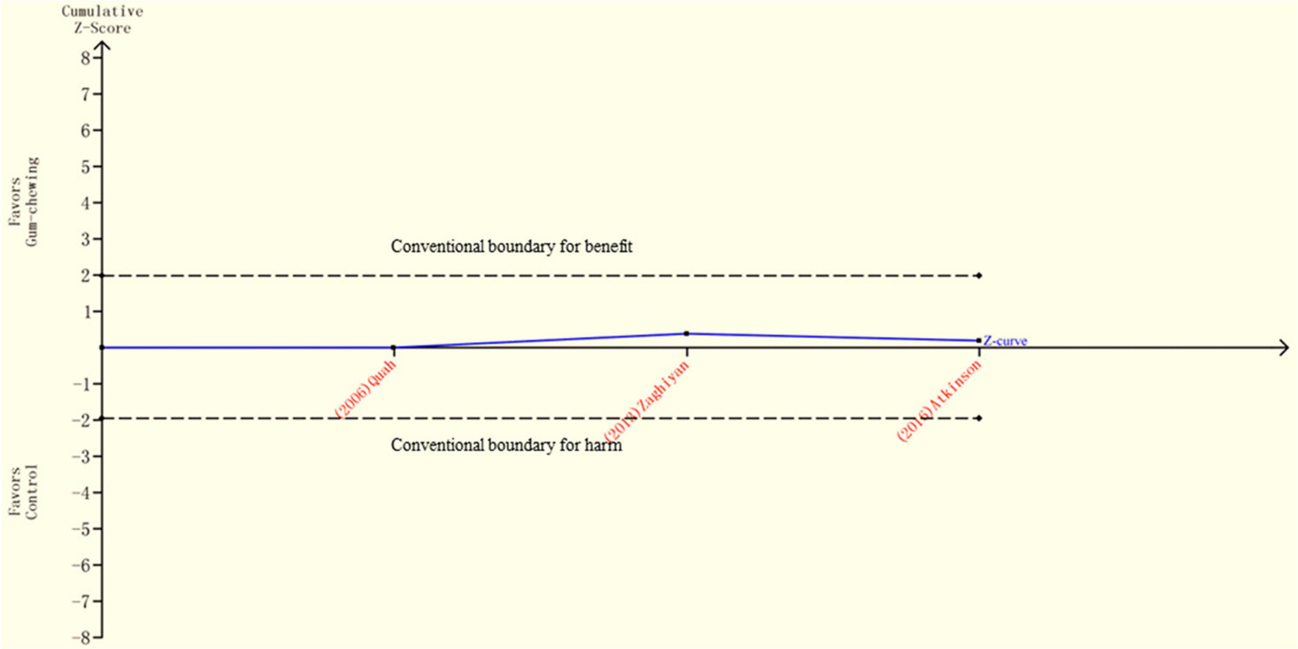

J. Trial sequential analysis of bleeding suggesting that 554 participants were accrued (required information size = 17478). The required information size was calculated using α = 0.05 (two-sided), β = 0.20 (power 0.80), a relative risk reduction of 20 %, a diversity of 0 %, and a control event proportion of 3.9 % obtained from the results of the meta-analysis. The trial sequential analysis exhibited that the cumulative Z-curve did not cross the conventional boundary for benefit and did not accrue the required information size. The trial sequential monitoring boundaries were ignored due to only 3.17 % of required sample size was reached in the trials of the meta-analysis. So we concluded that there is no sufficient evidence to detect the difference between the gum-chewing and control groups.

(*Continued*)

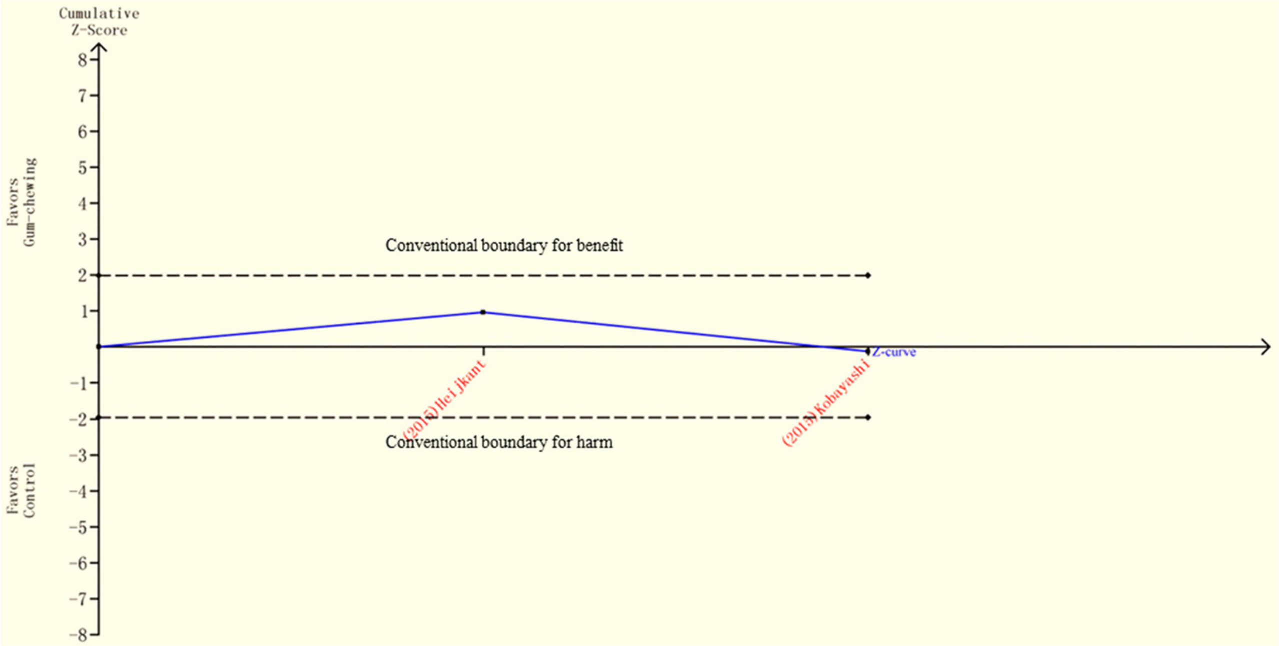

K. Trial sequential analysis of wound dehiscence suggesting that 157 participants were accrued (required information size = 58290). The required information size was calculated using α = 0.05 (two-sided), β = 0.20 (power 0.80), a relative risk reduction of 20 %, a diversity of 51 %, and a control event proportion of 3.9 % obtained from the results of the meta-analysis. The trial sequential analysis exhibited that the cumulative Z-curve did not cross the conventional boundary for benefit and did not accrue the required information size. The trial sequential monitoring boundaries were ignored due to only 0.27 % of required sample size was reached in the trials of the meta-analysis. So we concluded that there is no sufficient evidence to detect the difference between the gum-chewing and control groups.

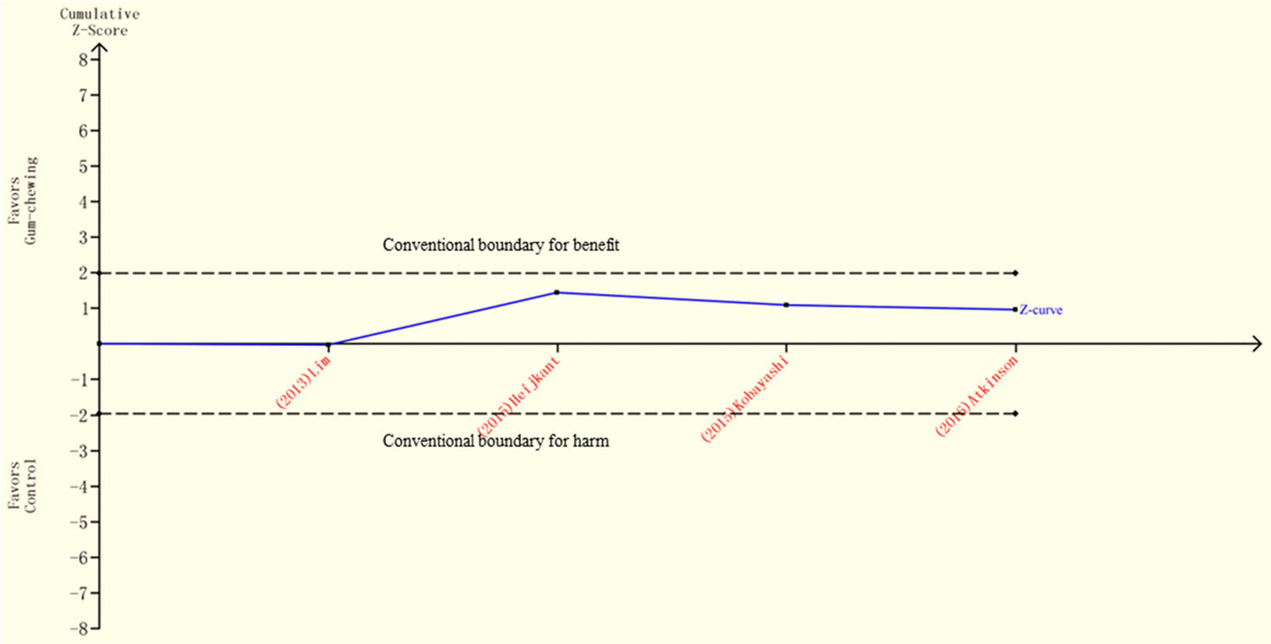

L. Trial sequential analysis of anastomotic leak suggesting that 714 participants were accrued (required information size = 58290). The required information size was calculated using α = 0.05 (two-sided), β = 0.20 (power 0.80), a relative risk reduction of 20 %, a diversity of 0 %, and a control event proportion of 4.66 % obtained from the results of the meta-analysis. The trial sequential analysis exhibited that the cumulative Z-curve did not cross the conventional boundary for benefit and did not accrue the required information size. The trial sequential monitoring boundaries were ignored due to only 4.89 % of required sample size was reached in the trials of the meta-analysis. So we concluded that there is no sufficient evidence to detect the difference between the gum-chewing and control groups.

(*Continued*)

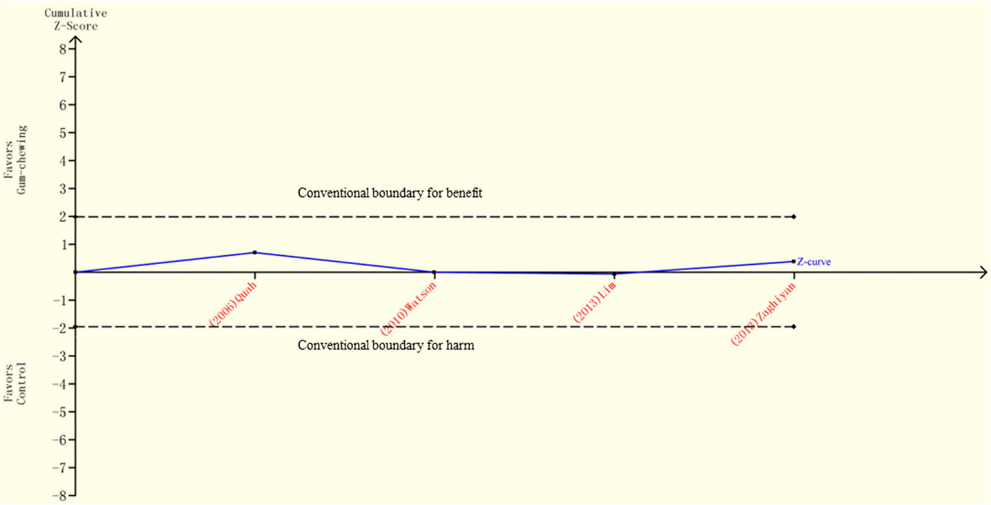

M. Trial sequential analysis of readmission suggesting that 396 participants were accrued (required information size = 15166). The required information size was calculated using α = 0.05 (two-sided), β = 0.20 (power 0.80), a relative risk reduction of 20 %, a diversity of 0 %, and a control event proportion of 4.43 % obtained from the results of the meta-analysis. The trial sequential analysis exhibited that the cumulative Z-curve did not cross the conventional boundary for benefit and did not accrue the required information size. The trial sequential monitoring boundaries were ignored due to only 2.39 % of required sample size was reached in the trials of the meta-analysis. So we concluded that there is no sufficient evidence to detect the difference between the gum-chewing and control groups. The trial by Schuster and colleagues (2006) was excluded due to it did not provide statistical information for performing trial sequential analysis.

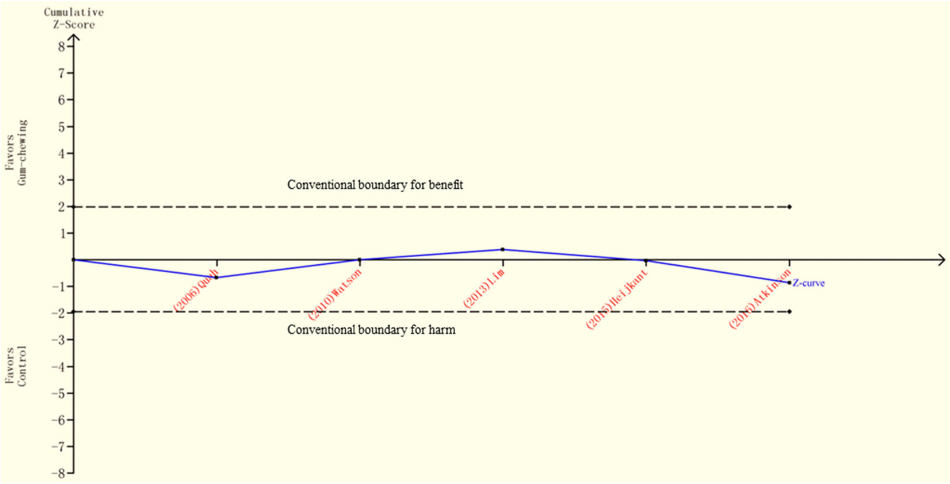

N. Trial sequential analysis of mortality suggesting that 802 participants were accrued (required information size = 15166). The required information size was calculated using α = 0.05 (two-sided), β = 0.20 (power 0.80), a relative risk reduction of 20 %, a diversity of 0 %, and a control event proportion of 0.74 % obtained from the results of the meta-analysis. The trial sequential analysis exhibited that the cumulative Z-curve did not cross the conventional boundary for benefit and did not accrue the required information size. The trial sequential monitoring boundaries were ignored due to only 0.84 % of required sample size was reached in the trials of the meta-analysis. So we concluded that there is no sufficient evidence to detect the difference between the gum-chewing and control groups. The trial by Bagena-Aponte and colleagues (2010) was excluded due to it did not provide statistical information for performing trial sequential analysis.

Supplementary Figure S4: Trial sequential analysis of dichotomous outcomes: overall complication A., other complications **B.**, postoperative ileus **C.**, nausea **D.**, vomiting **E.**, bloating **F.**, overall infection **G.**, wound infection **H.**, other infections **I.**, bleeding **J.**, wound dehiscence **K.**, anastomotic leak **L.**, readmission **M.**, and mortality **N.** We did not perform the trial sequential analysis to assess the complications related to gum-chewing and reoperation due to only one and two of all eligible trials respectively reported it. For meta-analysis of dichotomous data, a diversity-adjusted required information size and the trial sequential boundaries can be calculated and constructed respectively using a reasonable α, β, risk ratio reduction, and control event proportion.

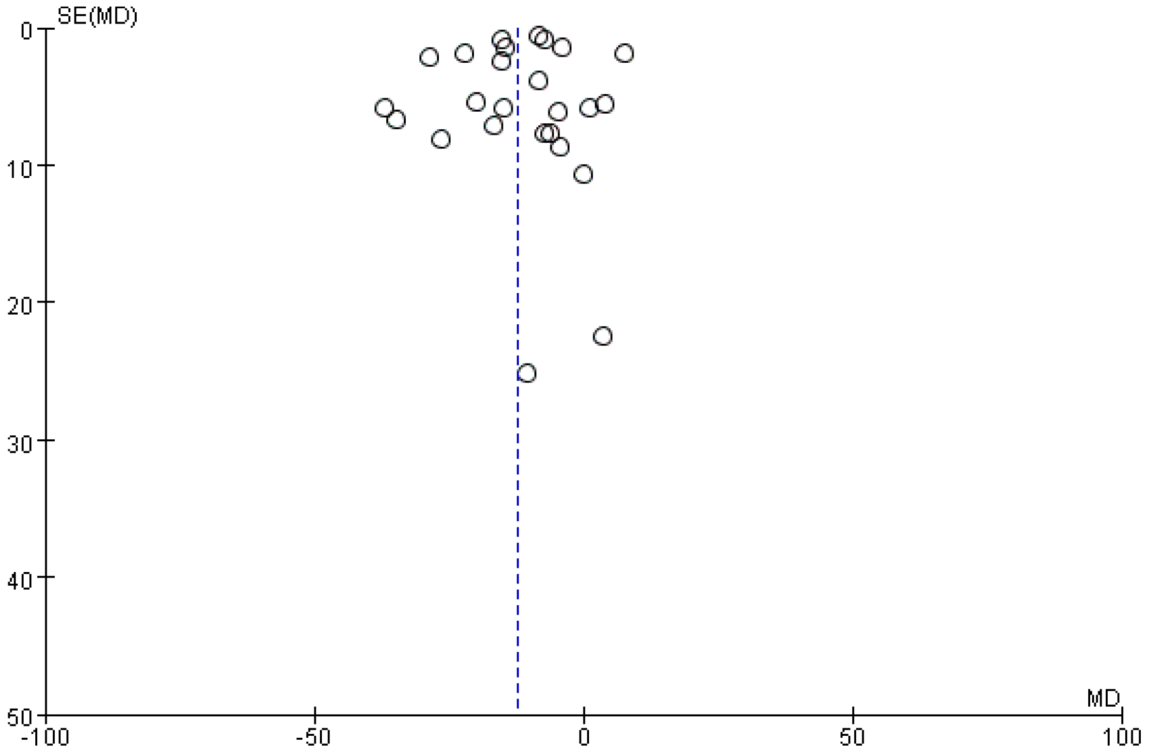

A. Time to first flatus

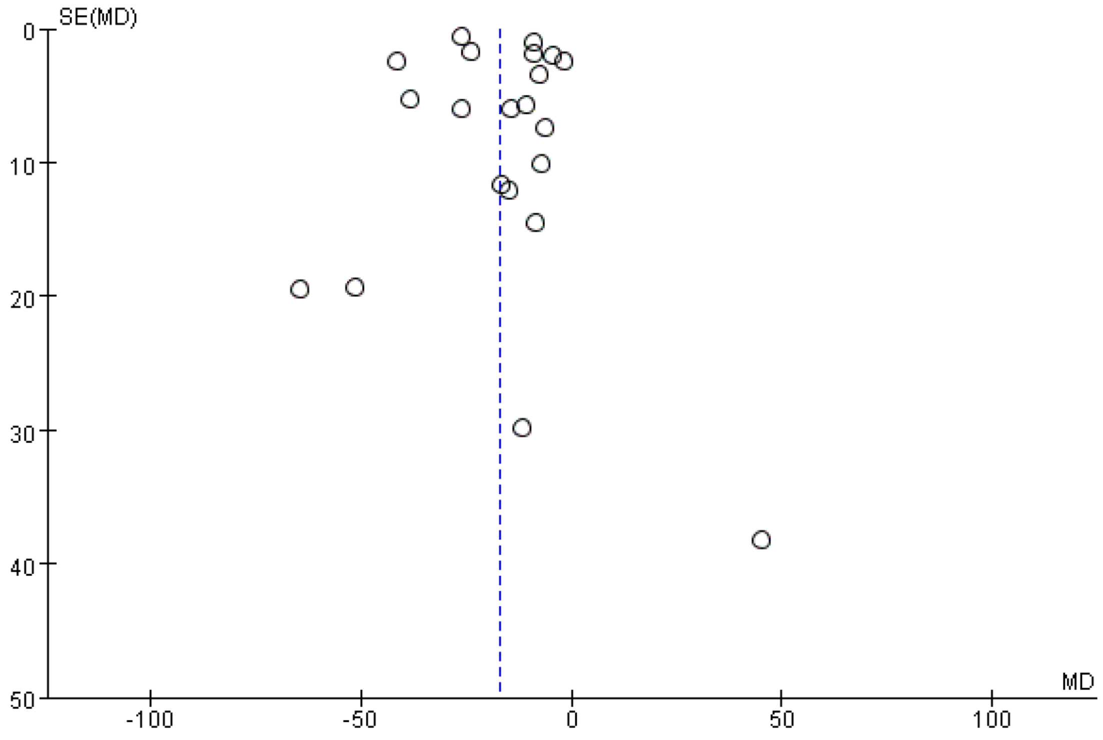

B. Time to first bowel movement

(*Continued*)

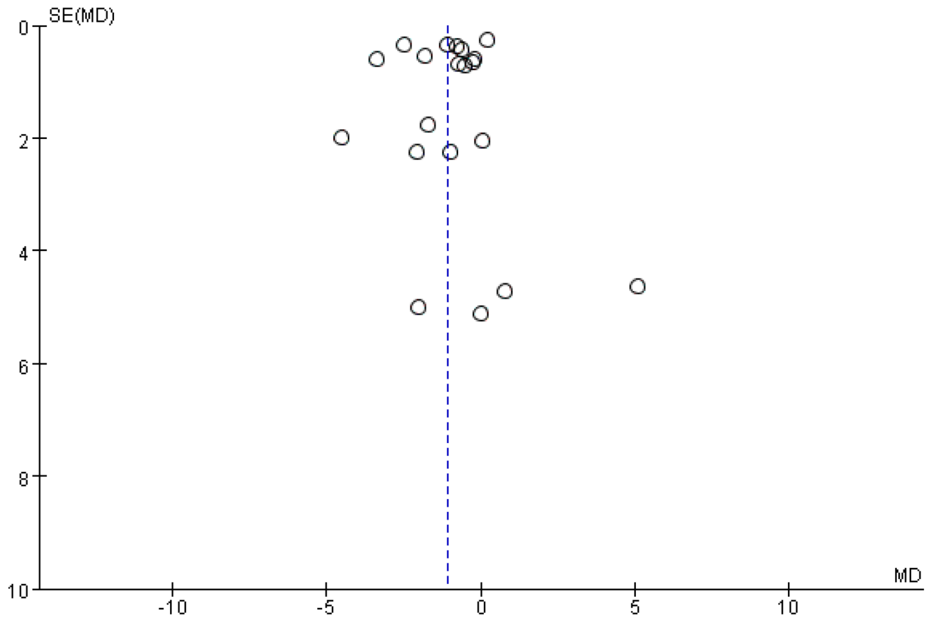

C. Length of hospital stay

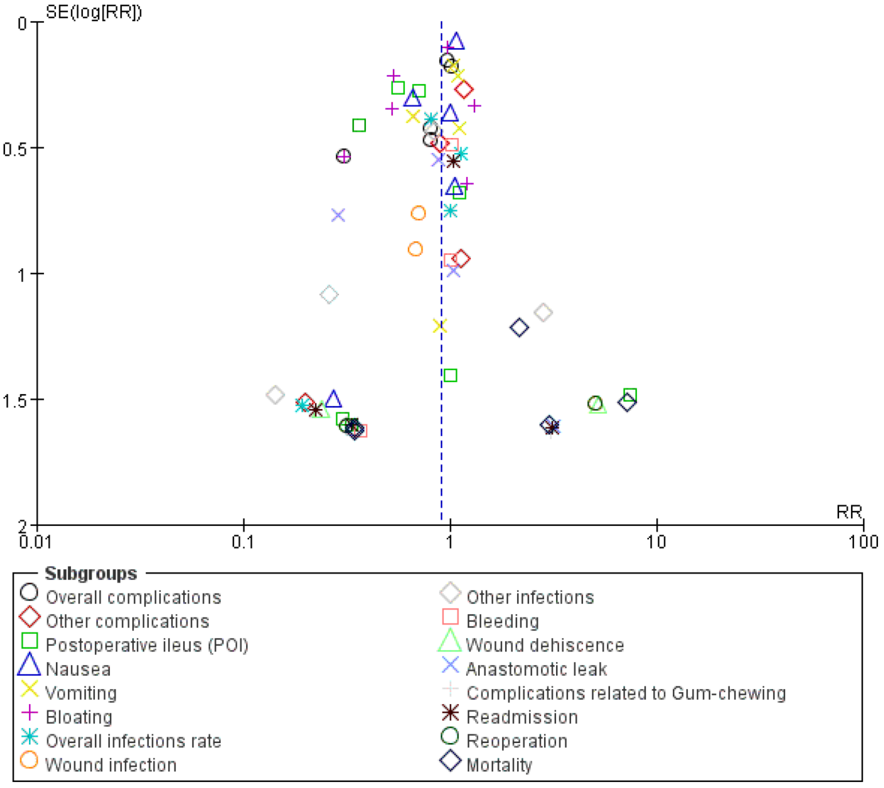

D. Secondary outcomes

Supplementary Figure S5: Funnel plots of publication bias: time to first flatus A., time to first bowel movement **B.**, length of hospital stay **C.**, and secondary outcomes **D.** The funnel plot for each outcome was drawn using the effect size (e.g. weighted mean difference [WMD] and risk ratio [RR]) and corresponding standard error, and a symmetric funnel plot indicates absence of publication bias.

Supplementary Table S1: GRADE evidence profile for all outcomes

See Supplementary File 1

Supplementary Table S2: Subgroup analyses concerning the different surgical procedures

See Supplementary File 2
