# Supplementary material for: Meta-analysis comparing chewing gum versus standard postoperative care after colorectal resection
Source: Oncotarget. 2016 Aug 31;7(43):70066–79. doi: 10.18632/oncotarget.11735 (PMC5342535; doi:10.18632/oncotarget.11735)
Supplement: Supplementary file 2 [file oncotarget-07-70066-s002.docx]

**Table.S2** Subgroup analyses concerning the different surgical procedures

| Procedures | Number of trials | *p* value for the  heterogeneity | Summary *MD* or *RR* (95% *CI*) | *p* value for the pooled estimates |
| --- | --- | --- | --- | --- |
| *Time to first flatus (hours)* |  |  |  |  |
| Laparoscopic colectomy | 1 | NA | -26.40 (-42.35, -10.45) | <0.01 |
| Open colectomy | 17 | <0.01 | -14.47 (-18.67, -10.27) | <0.01 |
| Hybrid method | 7 | <0.01 | -4.18 (-13.02, 4.66) | 0.35 |
| *Time to first bowel movement (hours)* |  |  |  |  |
| Laparoscopic colectomy | 1 | NA | -64.80 (-102.98, -26.62) | <0.01 |
| Open colectomy | 12 | <0.01 | -17.65 (-26.05, -9.24) | <0.01 |
| Hybrid method | 8 | <0.01 | -14.65 (-23.59, -5.71) | <0.01 |
| *Time to first bowel sounds (hours)* |  |  |  |  |
| Open colectomy | 3 | 0.62 | -6.39 (-6.69, -6.09) | <0.01 |
| Hybrid method | 1 | NA | 0.00 (-7.12, 7.12) | 1.00 |
| *Length of hospitalization (days)* |  |  |  |  |
| Laparoscopic colectomy | 1 | NA | -1.00 (-5.40, 3.40) | 0.66 |
| Open colectomy | 12 | <0.01 | -1.40 (-2.12, -0.67) | <0.01 |
| Hybrid method | 7 | 0.40 | -0.22 (-0.65, 0.21) | 0.31 |
| *Overall complication* |  |  |  |  |
| Open colectomy | 2 | 0.99 | 0.80 (0.43, 1.49) | 0.49 |
| Hybrid method | 3 | 0.89 | 0.97 (0.78, 1.21) | 0.80 |
| *Other complications* |  |  |  |  |
| Open colectomy | 1 | NA | 0.20 (0.01, 3.86) | 0.29 |
| Hybrid method | 3 | 0.90 | 1.09 (0.70, 1.70] | 0.71 |
| *Postoperative ileus* |  |  |  |  |
| Laparoscopic colectomy | 1 | NA | 0.30 (0.01, 6.62) | 0.45 |
| Open colectomy | 5 | 0.36 | 0.53 (0.32, 0.86) | 0.01 |
| Hybrid method | 2 | 0.53 | 0.75 (0.46, 1.23) | 0.25 |
| *Nausea* |  |  |  |  |
| Open colectomy | 4 | 0.69 | 0.79 (0.51, 1.21) | 0.28 |
| Hybrid method | 1 | NA | 1.07 (0.92, 1.25) | 0.38 |
| *Vomiting* |  |  |  |  |
| Open colectomy | 3 | 0.65 | 0.83 (0.49, 1.42) | 0.50 |
| Hybrid method | 2 | 0.89 | 1.06 (0.81, 1.38) | 0.70 |
| *Bloating* |  |  |  |  |
| Open colectomy | 5 | 0.08 | 0.66 (0.41, 1.06) | 0.09 |
| Hybrid method | 1 | NA | 0.97 (0.79, 1.18) | 0.74 |
| *Overall infection rate* |  |  |  |  |
| Open colectomy | 3 | 0.52 | 0.95 (0.42, 2.14) | 0.90 |
| Hybrid method | 1 | NA | 0.80 (0.37, 1.72) | 0.57 |
| *Wound infection* |  |  |  |  |
| Open colectomy | 3 | 0.40 | 0.87 (0.25, 2.96) | 0.82 |
| Hybrid method | 1 | NA | 0.68 (0.11, 4.03) | 0.67 |
| *Other infections* |  |  |  |  |
| Open colectomy | 3 | 0.23 | 0.63 (0.09, 4.20) | 0.63 |
| Hybrid method | 2 | 0.32 | 0.71 (0.32, 1.58) | 0.40 |
| *Bleeding* |  |  |  |  |
| Open colectomy | 1 | NA | 1.00 (0.16, 6.38) | 1.00 |
| Hybrid method | 2 | 0.55 | 0.94 (0.37, 2.35) | 0.89 |
| *Anastomotic leak* |  |  |  |  |
| Open colectomy | 2 | 0.18 | 0.63 (0.07, 5.62) | 0.68 |
| Hybrid method | 2 | 0.88 | 0.91 (0.36, 2.33) | 0.84 |
| *Readmission* |  |  |  |  |
| Open colectomy | 2 | NA | 0.33 (0.01, 7.70) | 0.49 |
| Hybrid method | 3 | 0.48 | 0.98 (0.37, 2.60) | 0.97 |
| *Mortality* |  |  |  |  |
| Open colectomy | 3 | 0.87 | 2.42 (0.36, 16.06) | 0.36 |
| Hybrid method | 3 | 0.27 | 1.02 (0.13, 7.91) | 0.98 |

MD, mean difference; RR, risk ratio; NA, not applicable
